# *Pseudomonas aeruginosa*: associated pathogenesis, epidemiology, resistance mechanisms, host response and emerging treatment strategies

**DOI:** 10.1007/s00203-026-04839-0

**Published:** 2026-04-24

**Authors:** Sheeba Sawant, Safura Shaikh, Timothy C. Baldwin, Aikaterini Karakoula, Ayesha Rahman

**Affiliations:** 1 Faculty of Science and Engineering, University of Wolverhampton, Wulfruna St, Wolverhampton, WV1 1LY UK; 2 Dentistry, School of Health Sciences, College of Medicine and Health, University of Birmingham, Edgbaston, Birmingham, B5 7EG UK

**Keywords:** *Pseudomonas aeruginos*a, Antibiotic resistance, Nosocomial, Pyocyanin, Biofilm, Virulence, Immune response, Alternative therapies, Quorum sensing

## Abstract

*Pseudomonas aeruginosa* is recognized as a critical-priority pathogen due to its major role in healthcare-associated infections (HAIs) and the rapid global rise of multidrug resistance. It is responsible for approximately 7.1–7.3% of HAIs worldwide and up to 23% of intensive care unit infections, specifically ventilator-associated pneumonia, where mortality can reach 32–42.8%. In chronic lung diseases, especially cystic fibrosis, *P. aeruginosa* commonly becomes the dominant pathogen in adulthood and contributes to progressive lung function decline. A major factor supporting long-term survival is biofilm formation, which can increase antimicrobial tolerance by up to 1000-fold compared with free-living bacteria. Virulence is further regulated through coordinated production of phenazines, rhamnolipids, exopolysaccharides, and quorum sensing signalling systems. The toxin pyocyanin, produced by 90–95% of clinical isolates, damages host tissues by disrupting cellular respiration, impairing ciliary activity, disturbing calcium balance, and promoting neutrophil apoptosis. During chronic infection, host defence mechanisms, including epithelial barriers, mucociliary clearance (~90% removal of inhaled particles), antimicrobial peptides, and immune cell responses, become less effective, while biofilms promote persistent inflammation and poor bacterial clearance. Increasing multidrug and carbapenem resistance (up to 47.6% in Europe and 32.8% in Asia) is driving interest in alternative therapies, including anti virulence agents, biofilm-disrupting enzymes, nanoparticles, bacteriophage therapy, and host-directed approaches targeting excessive inflammatory responses.

**Supplementary Information:**

The online version contains supplementary material available at 10.1007/s00203-026-04839-0.

## Background

A surge in antimicrobial resistance, particularly among Gram-negative bacteria, is one of the major concerns currently facing healthcare. Amongst the Gram-negative bacterial pathogens, *Pseudomonas aeruginosa* is a significant threat, mainly in immunocompromised and terminally ill patients (El Zowalaty et al. 2015; Su et al. 2025).

*P. aeruginosa* is commonly found in water and soil; it can also be isolated from humans, animals, and plants. This species can thrive in wet and humid environments and can metabolize a variety of organic compounds (Friedrich 2017). The ability of *P. aeruginosa* to survive in diverse environmental niches, from soil and water to human-associated habitats, not only underlines its ecological versatility but also sets the stage for its opportunistic colonisation in clinical settings, linking environmental persistence to heightened risks in healthcare. It is grouped as a Gram-negative, saccharolytic, non-spore-forming, aerobic bacillus, which measures between 0.5-0.8 µm in length and 1.5-3.0 µm in width. It is a lactose and glucose non-fermenting bacterium and is classed as a facultative anaerobe, which can utilize arginine and nitrate in the absence of oxygen, allowing it to multiply anaerobically with minimal nutrients (Arai 2011).

*P. aeruginosa* isolates can present multiple colony morphologies, such as mucoid and non-mucoid forms. Most colonies are flat and mucoid in appearance, which is due to the production of alginate, which is the major polysaccharide present in the exopolysaccharide (EPS) layer of *P. aeruginosa* biofilms (Moore and Flaws 2011a). The exopolysaccharide layer contributes to the biofilm structure making it impermeable to antimicrobial agents (Moradali et al. 2015). Most strains of *P. aeruginosa* bear a single, polar flagellum for swimming motility. *P. aeruginosa* is known to produce two soluble pigments: pyoverdine, a yellow-green/ brown pigment, commonly known as fluorescent pigment and pyocyanin, which results in blue-coloured, pigmented colonies (Moore and Flaws 2011a, b; Krell and Matilla 2024). Other rare water-soluble pigments, such as pyorubrin or pyomelanin, which result in red or brown-pigmented colonies have also been reported (Ngoshe et al. 2024).

*P. aeruginosa* infections in healthy individuals are self-limiting. However, contamination of hot tubs and pools can result in folliculitis and acute ocular infections in contact lens users (Subedi et al. 2018). Medical devices or equipment in active hospital settings are considered a reservoir of *P. aeruginosa* and can serve as a source of dissemination, leading to an outbreak of nosocomial infections (Khatoon et al. 2018). The morbidity and mortality rates linked with *P. aeruginosa* infections are increasing; however, the therapeutic options are limited, with the continuous emergence of antimicrobial-resistant strains. Given the serious impact of *P. aeruginosa* infections and the challenges in treating MDR strains, it is necessary to develop new and effective strategies to manage these infections. Furthermore, employing robust infection control practices and ensuring proper staff training in healthcare settings are key to preventing and limiting the spread of *P. aeruginosa* in hospitals. (Kubes and Fridkin 2019a).

This review outlines the key biological and virulence mechanisms, specifically antibiotic-resistant biofilm formation and environmental persistence, that drive multidrug-resistant (MDR) *P. aeruginosa* as a major nosocomial threat. It also highlights the limitations of current therapies and underscores the urgent need for new, effective antimicrobial strategies to improve patient outcomes.

## *P. aeruginosa-associated* infections

*Pseudomonas aeruginosa* is a major concern in healthcare systems due to hospital-associated infections, which are specifically challenging in intensive care units. Infections such as chronic obstructive pulmonary disease (COPD), pneumonia or cystic fibrosis (CF) are linked with a high rate of mortality and morbidity in individuals suffering from these infections (Winstanley et al. 2016; Fernández-Barat et al. 2017; Garcia-Nuñez et al. 2017; Wunderink and Waterer 2017; Kubes and Fridkin 2019b; Riquelme et al. 2020). The World Health Organization (WHO) has included *P. aeruginosa* in the critical category of the bacterial pathogen’s priority list for the exploration and development of new antimicrobials (Tacconelli et al. 2018; Botelho et al. 2019).

*Pseudomonas aeruginosa* and *Staphylococcus aureus* are commonly observed colonizers of the lungs in children with cystic fibrosis (CF). However, in adulthood, *P. aeruginosa* becomes the predominant pathogen driving the decline in lung function, as it outcompetes other microbes in the CF airway environment, which favours its growth. (Maurice et al. 2018a; Riquelme et al. 2020). A key factor to the persistence of *P. aeruginosa* is its ability to form biofilms; these communities provide strong protection against both antibiotics and host immune defences. Notably, they are not limited to surfaces and can also form free-floating aggregates within airway mucus, which makes treatment and bacterial clearance particularly challenging. (Belkova et al. 2025). Therefore, the prevalence of *P. aeruginosa* in adults with CF ranges from 31% in Ireland to 47% in the US (Reece et al. 2017). According to the UK Cystic Fibrosis 2020 Registry Annual Data Report, 31.9% and 17.8% of lung infection cases in CF patients are due to chronic and intermittent *P. aeruginosa* infections, respectively (Shaikh et al. 2021).

*P. aeruginosa* is a common pathogen in the lungs of patients with COPD and bronchiectasis (Garcia-Nuñez et al. 2017). Up to 15% of COPD patients can be colonised, uniquely those with advanced disease, mucoid strains, frequent exacerbations, prior ICU stays, active smoking, repeated antibiotics or corticosteroids, and coexisting bronchiectasis (Fujitani et al. 2011). Colonisation by *P. aeruginosa* may be transient, trigger acute flare-ups, or develop into a chronic infection, and is associated with reduced lung function, more frequent exacerbations, and increased mortality risk. In bronchiectasis, the bacterium promotes inflammation, airway obstruction, and a cycle of repeated infections that leads to long-term lung damage, contributing to high morbidity in healthcare settings. It can also cause pneumonia in individuals with pre-existing lung disease or weakened immunity, either through aspiration of oropharyngeal secretions or via bloodstream dissemination in immunocompromised patients (Kwok et al. 2025). Although using inhaled corticosteroids might raise the risk, it remains uncertain if *P. aeruginosa* directly makes the disease worse or exhibits a sign of already advanced lung problems (Eklöf et al. 2022)*.*

A fundamental problem in healthcare settings is the ability of *P. aeruginosa* to form biofilms on medical devices, implants, endotracheal tubes, urinary and vascular catheters (Engel and Balachandran 2009). It has been documented that 25% of burn patients’ wounds develop *P. aeruginosa* infections upon hospitalization (Schechner et al. 2011; Fujii et al. 2014). In addition, many patients develop urinary tract or lower respiratory tract infections during surgical treatment or following post-operative procedures. Patients with non-healing wounds or surgical sites are typically diabetic and due to failures in their nervous or circulation systems, they develop chronic wounds that often require amputation. These chronic wounds are ideal for colonisation by *P. aeruginosa* due to reduced blood circulation and the absence of skin, which weakens the immune response at the site, thereby increasing the prevalence of biofilm formation (Mulcahy et al. 2014; Miron et al. 2024).

Von Dossow et al. and Kang et al. documented that mechanical ventilation and respiratory failure were consistently associated with increased mortality in patients infected with *Pseudomonas* (Kang et al. 2005; von Dossow et al. 2005). A 2026 scoping review published in *Pathogens* (2026) confirmed that mechanical ventilation remains a coherent predictor of mortality, with rates for *P. aeruginosa* bloodstream infections reaching as high as 53% (Abdul Jabar et al. 2026). Likewise, a 2025 study recognized mechanical ventilation as a major independent predictor of 30-day mortality, with an adjusted odds ratio (aOR) of 7.33, suggesting that ventilated patients were over seven times more probable to die than those who were not (Maraolo et al. 2026). These modern results emphasise the earlier documentation by Von Dossow et al. and Kang et al., supporting that despite advances in treatment, the mechanical disruption and severity of respiratory failure remain to drive the maximum mortality risks.

*P. aeruginosa* is also a prominent source of nosocomial bacteremia (Sligl et al. 2015a). A surveillance project in the USA reported *P. aeruginosa* as the third most common Gram-negative pathogen isolated from hospitalized patients, causing nosocomial bacteremia (Schechner et al. 2011) and more recent evidence confirms that it remains among the third most frequent causes of bloodstream infections (BSIs), with mortality rates higher than most other Gram-negative bacteria (Ioannou et al. 2023). Its high prevalence in bloodstream infections is linked to *P. aeruginosa’s* remarkable environmental resilience, intrinsic ability to survive in moist hospital settings, and its frequent colonisation of medical equipment and indwelling devices. These features, combined with its capacity to form biofilms and rapidly acquire antimicrobial resistance, contribute to its prominence as a major cause of healthcare-associated bacteremia. (Ioannou et al. 2023). Nosocomial bacteremia accounts for roughly 7% of all hospital-acquired infections, with even higher prevalence in ICU settings (Ng et al. 2023). Septicemia caused by *Pseudomonas* has been related to skin lesions mentioned as ecthyma which differ from pustular lesions. Ecthyma lesions usually consist of a painless red macule; however, they can become further enlarged and develop into an elevated papule (Elmassry et al. 2020). This papule can then progress to form a hemorrhagic bulla which ruptures and forms a gangrenous ulcer with a black eschar bordered by an erythematous halo. (Huminer et al. 1987; Biscaye et al. 2017).

In the human gut, *P. aeruginosa* and *Candida albicans* are the most and second-most predominant microbiota, respectively, making natural interactions between them highly probable . During sepsis or catheter-related candidiasis, these organisms frequently translocate from the gut into the bloodstream. This migration is clinically concerning because mixed-species systemic infections are significantly more severe than those caused by a single organism (Phuengmaung et al. 2020).

Mixed-organism biofilms that form during bacteremia and fungemia further complicate the clinical picture. Particularly, *C. albicans* has been shown to interact synergistically with *P.aeruginosa*. Evidence from crystal violet staining suggests that these interactions enhance biofilm production and the resulting bacterial-fungal "collaboration" facilitated by gut leakage can lead to persistent, recurrent, or life-threatening infections (Phuengmaung et al. 2020).

Incidents of infective endocarditis (IE) caused by *P. aeruginosa* are uncommon and 90% of cases are associated with intravenous drug abuse (Tomoaia et al. 2022). *P. aeruginosa* IE involves right-sided heart valves leading to neurological complications such as brain abscesses, cerebral embolus, aseptic meningitis and ruptured mycotic aneurysm (Tomoaia et al. 2022). The high death rate (40%) associated with this infection, demands early diagnosis. The right-sided valve IE, displays a sub-acute clinical condition, correlated with failure of the right-hand side of the heart. However, left-hand-sided valve involvement presents as an advanced and complicated disease characterised by splenic abscesses, neurologic sequelae, systemic emboli, conduction abnormalities and congestive heart failure (Tomoaia et al. 2022).

The ocular infection most associated with *Pseudomonas* is keratitis; however, it may feasibly also cause endophthalmitis. Post-operative endophthalmitis is a serious complication that may result in sight loss or considerable visual impairment. (Kerr and Snelling 2009). Most ocular infections with this bacterium are due to contaminated, irrigating intraocular solutions, with poor prognosis given to patients. Post-surgery outbreaks are associated with contamination of the internal fluid pathways of a phacoemulsifer (Ramappa et al. 2012; Subedi et al. 2018). Corneal ulcers and their association with *P. aeruginosa* have been reported after surgeries or linked with trauma. Despite immediate treatment with intraocular antibiotics, endophthalmitis by *P. aeruginosa* often results in visual impairment (Eifrig et al. 2003).

Otolaryngologic infections due to *P. aeruginosa*, differ from relatively minor swimmer’s ear to malignant external otitis. Diabetic and elderly people are the most susceptible to developing malignant external otitis. The infection enters the deep tissue of the external auditory canal, due to cartilage defects which affect the base of the peripheral auditory canal. Consequently, there is progression in osteomyelitis of the temporal bone, with association of regions where the cranial nerves exit. Further development of osteomyelitis may lead to parietal or cerebral lobe brain abscesses. The symptoms vary from severe pain, purulent discharge, swelling of the external ear to presence of persistent tissue granulation (Bovo et al. 2012; Artono et al. 2025).

### Epidemiology

The global epidemiology of *P. aeruginosa* is characterised by its substantial contribution to healthcare-associated infections (HAIs) worldwide, with a prevalence estimated between 7.1% and 7.3% among all HAIs (Reynolds and Kollef 2021). However, a broader perspective reveals noteworthy regional and facility-based differences, predominantly concerning MDR strains. In ICUs globally, *P. aeruginosa* is a leading cause of infection, accounting for up to 23% of all ICU-acquired infections and frequently causing ventilator-associated pneumonia (VAP), where mortality rates can be as high as 32–42.8% (Reynolds and Kollef 2021; Schwartz et al. 2024).

The prevalence of MDR *P. aeruginosa* differs extensively across continents, with a pooled prevalence of carbapenem-resistant *P. aeruginosa* reported at 47.6% in Europe (highest) and 32.8% in Asia (lowest) in recent years (Ramatla et al. 2025). Furthermore, specific resistance phenotypes exhibit significant differences; for example, the prevalence of MDR *P. aeruginosa* in the Middle East and North Africa region ranges widely, from a low of 7.3% in some areas of Saudi Arabia and isolates in some US cohorts reported around 12.9% to a high of 64.5% in Lebanon (Al-Orphaly et al. 2021; Palavecino and Kilic 2021).

Similarly, in the United States, *P. aeruginosa* poses a major public health challenge, causing an estimated 32,600 healthcare-associated infections (HAIs) each year among hospitalized patients, which leads to significant direct medical costs (CDC data, 2025). Within the Veterans Health Administration system, *P. aeruginosa* bloodstream infections showed an average 30-day mortality rate of 23.3% (Hojat et al. 2024). *P. aeruginosa*’s most frequent reservoirs are medical devices and equipment, disinfectants, mops, sinks, food, and taps. Within 72 hours of hospital admission, the gastral carriage rates in patients have been reported to increase by up to 20 per cent (Iglewski BH. 1996). This phenomenon can be explained by the relocation of patients from other facilities and the introduction of vegetables, fruits, and plants into healthcare settings (CDC data, 2025).

The UK employs serious national surveillance to report *P. aeruginosa* bacteraemia. UK data reveal an erratic prevalence of *P. aeruginosa* bacteraemia, with an incidence rate of approximately 6.8 per 100,000 population in recent years (UK Health Security Agency 2025). Significantly, high-level genomic surveillance in the UK emphasises that these ICU infections are not acquired from the environment; instead, they are established and disseminated within the patient's own body (e.g., lungs to gut), highlighting the importance of host-associated factors such as the emergence of antibiotic resistance genes (Fisher et al. 2025).

Compared to earlier reports like Iglewski (1996), these recent figures highlight that the prevalence of *P. aeruginosa* infections has worsened over the years, reflecting increasing challenges in controlling this pathogen. These figures were derived from a multicenter point-prevalence study encompassing hospitals across multiple countries, reflecting global trends; however, differences in healthcare infrastructure and reporting practices may lead to regional variations in the prevalence and impact of *P. aeruginosa* infections.

As shown in Table 1, *P. aeruginosa* exhibits highly variable prevalence across different infection types, underscoring its status as a critical opportunistic pathogen. Specifically, the data show that the microbe is found in settings associated with tissue compromise and medical devices, with the highest reported prevalence in burn sites (33.9%) and VAP (20-36%) (A. Tchakal-Mesbahi; Miron et al. 2024). This pattern imposes the use of empiric antipseudomonal therapy in these high-risk critical care settings. Likewise, Table 1 highlights the organism's significant role in chronic disease, with a high prevalence of 60-80% in CF patients, underscoring the need for aggressive colonisation suppression. While general HAIs and UTIs exhibit lower rates (7.1% and 16.3%, respectively), these figures underscore the need for continuous local resistance surveillance to efficiently manage potentially MDR strains across all clinical environments (Reynolds and Kollef 2021).

**Table 1:** Illustrates the prevalence rates of *Pseudomonas aeruginosa*-associated infections, highlighting their occurrence in various clinical settings.

*Pseudomonas aeruginosa-*associated infections	Prevalence rate	Reference Publication Year	References
Healthcare-associated infections	7.1-7.3%	2023	(Reynolds and Kollef 2021a; Sathe et al. 2023)
COPD	4-15%	2020	(Jacobs et al. 2020)
Burn site infections	33.9%	2021	(Tchakal-Mesbahi 2021)
Urinary tract infections	16%	2021	(Reynolds and Kollef 2021b)
Ventilator-associated pneumonia	20-36%	2024	(Miron et al. 2024; Li et al. 2024)
Bacteraemia	7%	2021	(Reynolds and Kollef 2021b)
Cystic Fibrosis	60-80%	2025	(Belkova et al. 2025)

*P. aeruginosa* displays a diverse epidemiological profile, disproportionately altering vulnerable patient groups and occasionally leading to community outbreaks. In HIV patients, the infection risk is notably elevated; studies report the incidence of *P. aeruginosa* bacteremia is ten times higher than in the general hospital population. The independent risk factors identified in this patient population include severely low immune cell counts, specifically, counts less than 50 cells/ mm^3^). Furthermore, autopsy reports from 233 HIV-1 patients revealed that *P. aeruginosa* was responsible for bacterial bronchopneumonia reported in 16 out of 98 cases (Afessa et al. 1998; Vidal et al. 1999; Afessa and Green 2000).

The current epidemiology of *P. aeruginosa* infection in CF is defined by a major and steady drop in cases, which is a big change compared with past years, marking a dramatic reversal of historical trends across the 2015–2025 decade. Before the introduction of the highly effective CFTR modulator therapies (HEMTs) era (2015–2019), the chronic prevalence rate of *P. aeruginosa* remained consistently high, reported in the range of 40% to 50% across all age groups in major international registries (Foundation 2023). The broad adoption of HEMTs beginning around 2019 marked as the turning point, as the HEMTUS CF Foundation Patient Registry reported that the chronic prevalence dropped to approximately 27.6% in 2022. A similar pattern is further demonstrated by the latest UK CF Registry data 2024 report, which implies chronic infection in only 26.1% of people aged 16 and over, proving an accelerating decline in the carrier population (Report 2025). Studies showed rapid decrease with 30% to 50% of patients with established chronic infection can revert to an infection-free status within a year of starting HEMT treatment (Kapouni et al. 2023). Nonetheless, *P. aeruginosa* remains a formidable pathogen; the selective pressures introduced by HEMTs mean that the strains persisting in the remaining cohort often display highly adaptive traits such as mucoid phenotypes and elevated levels of multidrug resistance (Sheikh et al. 2023).

Developing on these observations, the epidemiology of *P. aeruginosa* reveals its significant competence to persist across diverse ecological niches and its role as a prominent nosocomial pathogen, often complicated by the development of antimicrobial resistance. (Morrison and Wenzel 1984). The focus of this epidemiological analysis is to examine the incidence of *P. aeruginosa* infections and to evaluate strategies to improve infection prevention and control.

### Antibiotic resistance in *P. aeruginosa* & its mechanisms

Infections caused by *P. aeruginosa* are life-threatening and have a wide-reaching impact on public health due to its survival, resistance, and adaptation to multiple classes of antibiotics. Strains of *P. aeruginosa* have been reported to develop resistance to nearly all classes of antibiotics commonly used, including cephalosporins, aminoglycosides, carbapenems, and fluoroquinolones (Botelho et al. 2019). In the USA, around 13% of *P. aeruginosa* infections are caused by multidrug-resistant strains and hospital-associated infections are thought to cause approximately 51,000 cases each year (Poole 2011; editorial team 2013). The treatment of *P. aeruginosa* infections is becoming increasingly challenging due to acquired or mutational antibiotic resistance. (Driscoll et al. 2007). The selection of antibiotics for *P. aeruginosa* varies according to factors such as resistance patterns, site of infection, patient history and allergies, and availability of antimicrobial agents. The ever-increasing antimicrobial resistance (AMR) amongst Gram-negative pathogens has been a major concern for the treatment of infections in the healthcare system (Peña et al. 2009). Between April 2020 and March 2021, the National Health Service, UK reported 4,285 cases and 39.0% of which were healthcare-associated (Public Health England, 2020).

Eight categories of antibiotics are primarily used against *P. aeruginosa* infections, including carbapenems (meropenem, imipenem), cephalosporins (cefepime, ceftazidime), aminoglycosides (gentamicin, amikacin, netilmicin, tobramycin), fluoroquinolones (levofloxacin, ciprofloxacin), fosfomycin, polymyxins (polymyxin B and colistin), penicillin with β-lactamase, and piperacillin and ticarcillin in combination with tazobactam or clavulanic acid. (Bassetti et al. 2018).

The ever-increasing diversity and complexity of resistance mechanisms in *P. aeruginosa* have direct clinical implications, specifically in controlling empirical therapy and enhancing patient outcomes (Tchakal-Mesbahi 2021). Modern international and national guidelines highlight modifying initial empirical treatment to local resistance epidemiology, individual risk factors for MDR strains, and the site of infection. For severe hospital-acquired or VAP infections where MDR *P. aeruginosa* is suspected, combination therapy, classically a β-lactam with proven antipseudomonal activity (such as ceftazidime, cefepime, piperacillin–tazobactam, or a carbapenem) plus an aminoglycoside or fluoroquinolone, is frequently prescribed to increase the probability of early effective coverage (Ng et al. 2023). In settings with high prevalence of carbapenem-resistant strains, newer β-lactam/β-lactamase inhibitor combinations such as ceftolozane–tazobactam or ceftazidime–avibactam are recommended due to improved stability against AmpC and ESBL-mediated hydrolysis (Abdel-Fatah et al. 2024). These recommendations highlight the clinical reality that efflux pump overexpression, porin loss (e.g., OprD), and β-lactamase production directly restrict treatment options and impose rapid susceptibility testing and prevent further resistance development (Mohanty et al. 2020).

*P. aeruginosa* exhibits intrinsic resistance mechanisms to several classes of these antibiotics due to its outer membrane’s limited permeability and a range of efflux systems affecting antibiotics such as aminoglycoside, polymyxins, quinolones and β-lactams (Hancock 1998; Elfadadny et al. 2024). This wide variety of mechanisms to overcome antibiotic treatment is due to this microorganism's dissemination in aquatic habitats, which create a reservoir of microbes carrying a wide spectrum of antibiotic resistance genes (Vaisvila et al. 2001; Bassetti et al. 2018).

It is widely accepted that improper initial antibacterial therapy adversely impacts patient outcomes. The significance of appropriate therapies against blood-borne infections caused by *P. aeruginosa*, has been discussed in detail previously. (Micek et al. 2005). In this report, elevated mortality rates (30.7% v/s 17.8%) were noted in patients who failed to receive appropriate primary antimicrobial therapy. Moreover, initial treatment with various drug combinations, demonstrated improved activity in comparison to monotherapy against *P. aeruginosa*, suggesting the predominance of AMR (Leibovici et al. 1998; Kollef et al. 1999; Micek et al. 2005; Elfadadny et al. 2024).

Efflux pumps are proteins that are embedded in the bacterial plasma membrane. Penetration of noxious agents through the bacterial cell wall, reaching the periplasm or cytoplasm can be recognized by these efflux pumps and thereby be removed from within the bacterial cell out into the external environment (Amaral and --Molnar 2012; Amaral et al. 2014; Elfadadny et al. 2024). The efflux pump systems associated with the antimicrobial resistance in *P. aeruginosa*, belong to the resistance-nodulation-division (RND) family (Livermore 2001, 2002; Li and Nikaido 2009). Out of 12 RND efflux systems, four such as the MexAB-OprM, MexCD-OprJ, MexEF-OprN, and MexXY-OprM, are known to assist in resistance to several antibiotics (Poole 2011; Bassetti et al. 2018). MexCD-OprJ and MexEF-OprN are involved in acquired resistance, whereas MexAB-OprM and MexXY-OprM are implicated in acquired and natural resistance (Bassetti et al. 2018). Acquired resistance is observed due to the induction of antibiotic pressure, which results in mutations within regulatory systems that lead to overexpression of efflux systems, potentially conferring resistance to all categories of antibiotics, except polymyxins (Strateva and Yordanov 2009; Mazza et al. 2025). Resistance to multiple antibiotic classes may be triggered by a single exposure to an antibiotic that serves as a substrate for efflux systems. Quinolones are substrates for all known efflux systems and can act as triggering factors, causing cross-resistance to the majority of antibiotics commonly used for the treatment of P. aeruginosa infections, including β-lactams and aminoglycosides (Masuda and Ohya 1992; Poole 2005; Mesaros et al. 2007a; Mazza et al. 2025).

Several mechanisms can induce membrane impermeability, such as lipopolysaccharide alterations, inactivation of enzymatic complexes which are essential for transporter activity, and modification of membrane proteins associated with substrate uptake (Lambert 2002; Strateva and Yordanov 2009). In addition, the OprD porin - transmembrane protein - is recognized to assist in the absorbency of imipenem and to a lesser degree, meropenem. However, alteration in OprD structure and/or a decline in its expression may reduce its sensitivity to imipenem (Pechère and Köhler 1999; Köhler et al. 1999). Moreover, OprD porin modification is correlated with overexpression of efflux pumps, thereby conferring higher resistance to imipenem and other antibiotics, such as aminoglycosides and quinolones. (Mesaros et al. 2007b; Amisano et al. 2025).

β-lactamase inhibitors (BLI) such as tazobactam, clavulanic acid and sulbactam are unable to inhibit the AmpC cephalosporinase, encoded by the wild-type *P. aeruginosa* strain (Sligl et al. 2015b). The low expression of AmpC cephalosporinase, coupled with multiple efflux systems and restricted membrane permeability, provides resistance to 1^st^ and 2^nd^ cephalosporin generations (C1G, C2G), cephamycin (C3G), ceftriaxone and ceftriaxone, aminopenicillins and in combinations with BLI, and ertapenem and carbapenem (Öncül et al. 2014; Lund-Palau et al. 2016). The β-lactamases are restricted-spectrum, class A enzymes that are plasmid-encoded. However, several acquired extended spectrum β-lactamase (ESBL) enzymes have been identified in *P. aeruginosa,* which can target a wider range of β-lactams, as well as carbapenems, are becoming of major concern (Zhou et al. 2010). ESBL *Enterobacteriaceae* enzymes such as CTX-M, TEM and SHV have all been shown to be expressed in *P. aeruginosa*, due to horizontal gene transfer (Table 2) (Chanawong et al. 2001; Mohanty et al. 2020; Castanheira et al. 2021a, b). These enzymes provide resistance to ceftazidime, cefpirome, cefepime, ureidopenicillins, carboxypenicillins and aztreonam (Cristina et al. 2009; Bassetti et al. 2018), and are inhibited by tazobactam and clavulanic acid (Pechère and Köhler 1999; Köhler et al. 1999; Castanheira et al. 2021a).

**Table 2:** Enzymes/ efflux pump responsible for antibiotic resistance (Mohanty et al. 2020; De Gaetano et al. 2023)

Sr.No.	Enzymes/ efflux pump responsible for resistance (Mode of action)	Antibiotics
1.	Production of β-lactamase	Ticarcillin/clavulanate
2.	Overexpression of *MexC-MexD-OprJ* operon	Chloramphenicol and norfloxacin
3.	Overexpression of *MexEF-OprN* operon Expression of *MexC-MexD-OprJ* operon	Ciprofloxacin
4.	Loss of porin channels in the outer membrane, expression of OprD and secreting carbapenem-hydrolysing metalloenzyme	Carbapenem (imipenem and meropenem)
5.	Inactivation of aminoglycoside enzyme, ribosomal methyl group transferase enzyme	Carbenicillin and tobramycin
6.	Overexpression of *MexAB-OprM* operon	Gentamicin
7.	Inhibition of *MexXY-OprM* activity	Tigecycline
8.	Overexpression of *MexAB-OprM* efflux pump due to the NalB mutation	Cephalosporin
9.	Activation of *MexXY-OprM* efflux pump	Tetracyclines
10.	Inactivation of *MexC* operon	Ofloxacin
11.	Modification in the LPS layer	Polymyxin E (colistin)
12.	DNA gyrase topoisomerase IV activity	Fluroquinolones
13.	Expression of *MexEF-OprN* efflux pump due to mutation of NfxB, NfxC and NalB	Quinolones
14	OprD porin transmembrane protein	Imipenem, meropenem
15	Alteration in OprD structure	Imipenem
16	Enzymes CTX-M, TEM, SHV expressed due to horizontal gene transfer	Ceftazidime, cefpirome, cefepime, ureidopenicillins, carboxypenicillins and aztreonam
17	Class B enzymes, IMP-1 activity	Cephalosporins, meropenem and imipenem

Class B enzymes found in *P. aeruginosa*, such as the enzyme IMP-1 can hydrolyze β-lactam antibiotics such as cephalosporins, meropenem and imipenem (Minami et al. 1996). Fortunately, piperacillin and aztreonam are less affected by IMP-1 activity (Nordmann and Guibert 1998). Class D enzymes consist of several oxacillinases, which are responsible for extended-spectrum β-lactamases observed within this bacterium (Lister et al. 2009). Epidemiologic data, coupled with microbiological evidence, is essential to formulate an approach to combat the antibiotic resistance mechanisms of *P. aeruginosa*. In order to cause nosocomial infections, *P. aeruginosa* must successfully infect numerous patients and thus become exposed to a wide variety of antimicrobial therapies, this exposure to a range of antibiotics, causes the bacteria to develop multiple antibiotic resistance mechanisms (Castanheira et al. 2021b).

The diverse resistance mechanisms exhibited by *P. aeruginosa* such as enzymatic inactivation, altered membrane permeability, active efflux, and mutation-driven adaptation, emphasize its capacity to survive antimicrobial pressures in clinical settings. Importantly, these mechanisms do not operate in isolation but are tightly coordinated through complex regulatory networks that enable the bacterium to sense, interpret, and respond to external stressors. Among these, two-component systems (TCSs) play a central role by integrating environmental cues, involving antibiotic exposure, and coordinating appropriate transcriptional responses. The following section explores the regulatory interplay between the structure, function, and significance of TCSs in mediating *P. aeruginosa* virulence and antimicrobial resilience*.*

### Two-component systems (TCS)

**Fig. 1:**
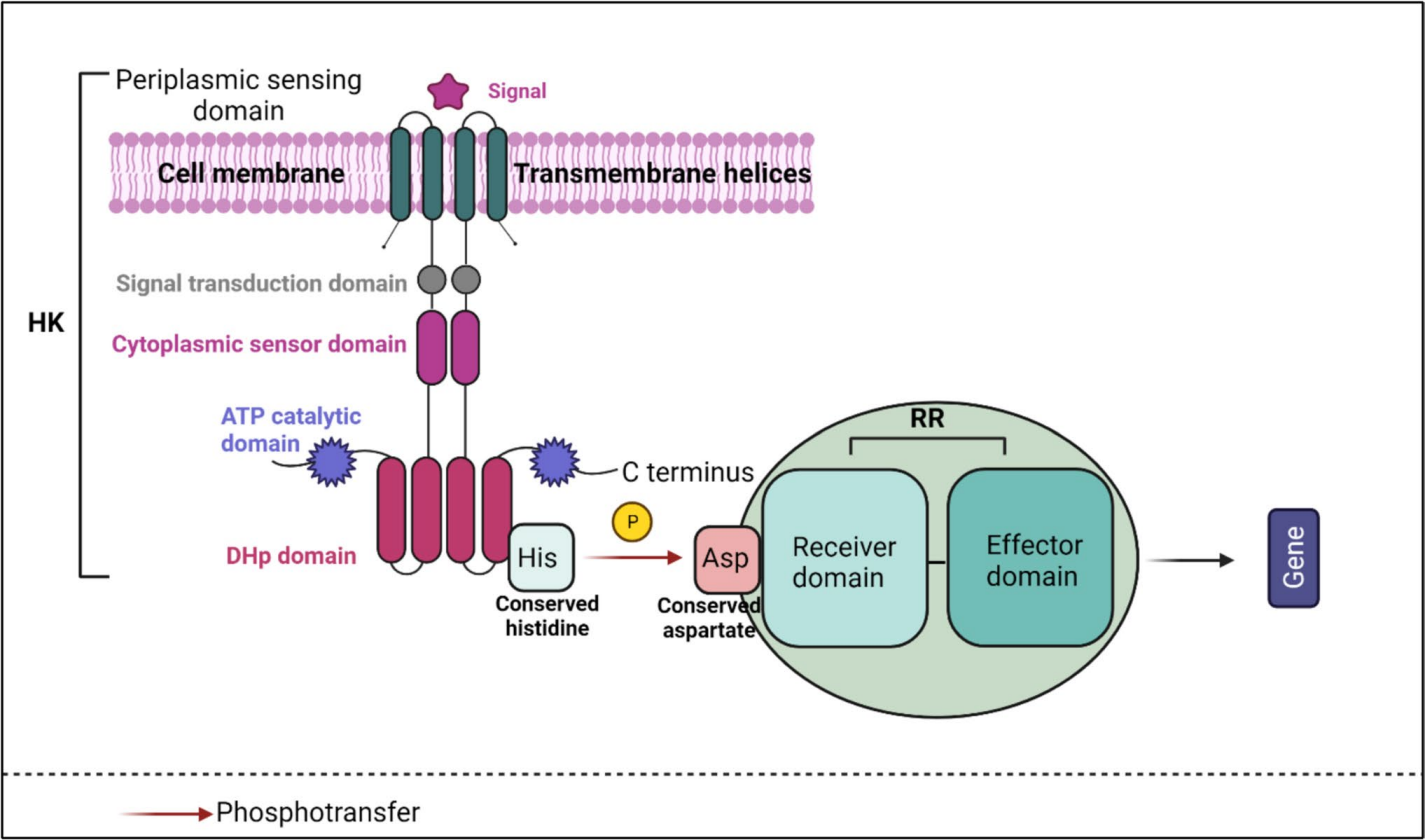
A diagrammatic representation of a two-component signaling system. The sensor Histidine kinases sense the external stimuli, using the sensing domain that is attached to the signal transduction domain, a cytoplasmic sensor domain, an ATP catalytic domain, a DHp domain. The histidine of HK transfers the phosphate to the aspartate of the receiver domain of its response regulator (RR). The effector domain of the phosphorylated RR attaches to its target and controls gene expression. Adapted from (Sultan et al. 2021)

The versatility of *P. aeruginosa* in virulence traits and resistance patterns is associated with its large genome and primary genes (Stover et al. 2000; Poulsen et al. 2019). The genome of this bacterium consists of multiple two-component systems (TCSs) and various regulatory genes (Rodrigue et al. 2000). To identify and respond to signals in distinct environments, the TCS functions through a signal-response coupling mechanism (Fig. 1), and these systems regulate the expression of virulence factors in *P. aeruginosa.* (Francis et al. 2017; Sultan et al. 2021).

It is essential to understand how P. aeruginosa responds to environmental stimuli via the two-component system during the infectious period to broaden our understanding of its pathogenesis. Understanding the role of two-component systems in virulence can allow us to explore novel antimicrobial agents against the AMR strains of *P. aeruginosa* (Krell and Matilla 2024). The endurance and adaptation of *P. aeruginosa* to hostile surroundings can be attributed to these diverse TCS-regulated response mechanisms. (Francis et al. 2017; Sultan et al. 2021).

The two-component systems that are the leading mediators of signal transduction consist of a sensor histidine kinase (HK) and its cognate response regulator (RR), which directs the signal transduction pathways (Stock et al. 2000; Krell and Matilla 2024). These systems play an important role in detecting and responding to various external and internal stimuli, such as gas and ion concentrations, nutrient levels, redox states, cell density, and temperature. (Goodman et al. 2009).

The sensor HK is composed of multidomain structures, predominantly comprised of a periplasmic sensing domain that is in control of recognising distinctive signals, followed by the signal transduction domain and the cytoplasmic sensor domain, ATP (adenosine triphosphate) catalytic domain and the DHp, dimerization histidine phosphotransferase domain. (Stock et al. 2000; Raghavan and Groisman 2010; Sultan et al. 2021).

The HK sensor induces histidine autophosphorylation by transferring phosphate from aspartate to its cognate RR. Thereafter, the effector domain undergoes conformational modifications allowing it to bind with DNA, thus triggering changes in gene expression.

**Fig. 2:**
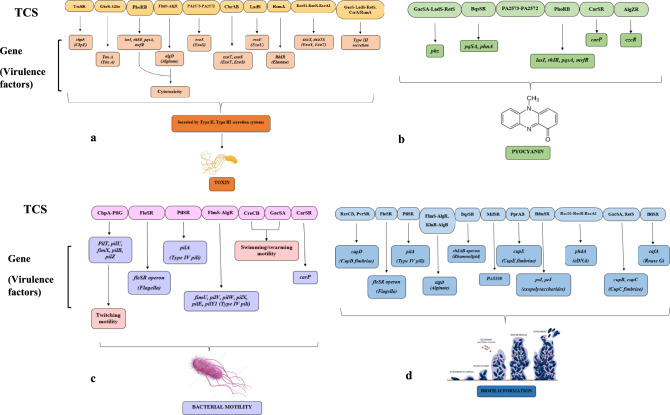
Conceptual representation of two-component systems (TCS) regulating major virulence traits in *P. aeruginosa*. The panels illustrate how TCS regulate different pathogenic processes: **a** Toxin production, where systems such as TtsSR, GtrS-Gltr, PhoRB, FlmS-AlgR, PA2573-PA2572, CbrAB, LadS, RsmA, RocS1-RocR-RocA1, and GacS-LadS-RetS/CsrA/RsmA regulate virulence genes including cbpE, toxA, algD, exoS, exoT, exoU, and rhlR, resulting in cytotoxic effects via Type II and Type III secretion mechanisms. **b** Pyocyanin synthesis, controlled by TCS such as GacS-LadS-RetS, BqsSR, PA2573-PA2572, PhoRB, CarSR, and AlgZR, modulates the expression of phz, pqsA/phnA, and quorum-sensing genes (lasI, rhlR, pqsA, mvfR). **c** Motility, including swimming, swarming, and twitching, is regulated by TCS like ChpA-PilG, FleSR, PilSR, FlmS-AlgR, CreCB, GacSA, and CarSR, which control pilus- and flagella-associated genes (pil, fim, flg, fli). **d** Biofilm development, governed by TCS such as RcsCB-PvrSR, FleSR, PilSR, FlmS-AlgR, BqsSR, MifSR, PprAB, BfmSR, RocS1-RocR-RocA1, GacSA-RetS, and BfiSR, coordinates the expression of adhesion, exopolysaccharide, fimbrial, and extracellular DNA genes (cup, psl, pel, algD, phd4, cafA), facilitating biofilm maturation

Among the Gram-negative bacteria, *P. aeruginosa* has the highest number of two-component systems, comprising of 63 histidine kinases and 64 response regulators, and other pathogens such as *Klebsiella pneumoniae* HS11286 (32 HK, 32 RRs), *Escherichia coli* K12 (28 HKs, 32 RRs), and *A. baumanni* XH386 (14 HKs; 15 RRs), depicting complex TCSs control strategies of *P. aeruginosa.* (Harmsen et al. 2010; Pinglei et al. 2012; Fang et al. 2016; Sultan et al. 2021). Some of the TCSs associated with key virulence factors of *P. aeruginosa*, such as motility (flagella, pili), biofilm formation, pyocyanin pigment production, phospholipases, cytotoxins, proteases and elastases are shown in Fig. 2.

## Pathogenesis

*P. aeruginosa* pathogenesis is driven by an organized network of virulence factors such as motility, biofilm development, exopolysaccharide production, pigment synthesis, and quorum sensing that enable the bacterium to initiate infection, adapt to host environments, and persist during chronic disease. Previous studies (Smith et al. 2006) have revealed that *P. aeruginosa* isolates from acute infections differ phenotypically from those from chronic infections. Isolates from acute infection express a variety of virulence factors; however, the isolates from chronic diseases are observed to downregulate virulence mechanisms such as the type 3 secretion system and display an absence of inflammatory bacterial characteristics, such as pili and flagella (Hogardt and Heesemann 2010). In addition, the isolates from chronic infections easily form biofilms due to overexpression of exopolysaccharide alginate, which results in the production of a mucoid strain (Gellatly and Hancock 2013a). Clinical data of key virulence factors contributing to pathogenesis, mutations in isolates and differences in genotypes/phenotypes of isolates, can all be used to explore the contribution of specific virulence factors to human disease.

### Motility

The pathogen possesses key virulence-associated appendages, including a single polar flagellum and multifunctional type IV pili. They provide the ability to adhere to cell membranes or other surfaces, as well as motility. The major site for attachment is the respiratory tract, via the glycolipid asialoganglioside M1 on epithelial cell surfaces, resulting in a strong NFxB inflammatory response via TLR5 and Ipaf signaling molecules (Driscoll et al. 2007; Rasamiravaka et al. 2015; Sendra et al. 2024).

Type 4 pili (T4P) are classified into different classes: Type VIa (T4aP), Type VIb (T4bP), and Tad (tight adherence pili). Tad pili are notably smaller compared to T4aP and T4bP. *P. aeruginosa* is unique in having all three types of pili (Bernard et al. 2009). All strains of *P.aeruginosa* possess T4aP and Tad pili, while strains with the pathogenicity island PAPI-1 also have T4Bp (Carter et al. 2010). Each T4P class assembles via a distinct system: T4bP uses the PilD prepilin peptidase used by T4aP, while the Tad system has its own prepilin peptidase, FppA. Unlike T4aP, which is associated with motility due to the PilT ATPase required for pilus retraction, both T4bP and Tad pili generally lack PilT and thus are not primarily involved in motility. However, some T4bP may exhibit retraction capabilities under certain conditions (de Bentzmann et al. 2006; Craig and Li 2008).

#### Types of Motilities

*P. aeruginosa* expressing T4aP exhibits pilus-mediated twitching motility in conditions equivalent to the surface viscosity of 1% agar. However, the distance travelled via twitching motility varies depending upon many intrinsic and extrinsic factors, namely retraction rates, number of pili produced, hydrophobicity of surface, viscosity and nutrient composition (Semmler et al. 1999; Huang et al. 2003). The combination of T4aP, a carbon source such as glucose, glutamate or succinate with a less viscous substrate (equivalent 0.4% to 0.7% agar) results in swarming motility, by pilus-related chemotaxis. Mutation of the gene encoding for the ChpA protein, can decrease/modulate this swarming motility (Huang et al. 2003; Leech and Mattick 2006; Murray and Kazmierczak 2008).

*P. aeruginosa* expressing T4aP presents two types of motility i) crawling motility, explained as cell bodies which are parallel to the surface in alignment with slow movement which are not expected to change the direction of their movement. ii) walking motility, explained as cell bodies which are aligned at right angles to the surface plane with attachment via pili extended from the surface proximal pole, with faster paced motility and expected to change direction while moving (Gibiansky et al. 2010; Conrad et al. 2011).

### Biofilm formation

Bacteria can exist in two different forms: the planktonic form, which is free-floating or the sessile form (adhered to a surface), and these can exist simultaneously. The sessile state is also referred to as a biofilm, formed of a consortium of bacteria encapsulated in a self-produced, hydrated polymeric matrix termed an extracellular polymeric matrix (EPS) (Donlan 2001; Lee and Yoon 2017). Biofilm formation is an attachment process of microorganisms on a surface, and in conjunction with the production of an extracellular polymer/matrix allows for better attachment to the surface, and results in the alterations in gene expression and phenotypic characteristics Biofilm formation can occur both on biotic or abiotic surfaces including medical devices including catheters, contact lenses, pacemakers and heart valves, which are significant risk areas for infection, despite extreme caution and sterile environments being used. Other microbial infections due to biofilm formation include endocarditis, cystic fibrosis, osteomyelitis, and prosthetic joint infections (Donlan 2001; MartÃ­nez and Vadyvaloo 2014; Khatoon et al. 2018; Tuon et al. 2022)

Biofilms provide high levels of protection to microorganisms and contribute to resistance towards antimicrobial therapies. The planktonic form is reported to be 1000 times more susceptible to antimicrobial treatments as compared to the sessile form (Khatoon et al. 2018). Biofilm formation is an adaptive resistance mechanism, usually observed with high-density bacterial growth. Biofilms protect against antibiotics, altered pH, osmolarity, temperature fluctuation, nutrients scarcity and host immune response (Ma et al. 2009; Kang and Kirienko 2018; Khatoon et al. 2018; Higazy et al. 2024).

A biofilm includes a matrix, which accounts for 90% of the biofilm mass and provides adhesion to biotic and abiotic surfaces. The matrix also facilitates cell-to-cell communication. There are four key steps in biofilm formation: (i) initial attachment (ii) colonisation and biofilm formation (iii) biofilm maturation (iii) dispersal of the biofilm. Planktonic bacteria initially attach to the surface via physical forces and cell-surface interactions. Following this, the bacteria adhere irreversibly and become embedded in a matrix of extracellular polymeric substances, forming bacterial aggregates (MartÃ­nez and Vadyvaloo 2014; Rasamiravaka et al. 2015; Lee and Yoon 2017; Thi et al. 2020). The three major exopolysaccharides involved in biofilm development are Psl, Pel and alginate.

There are two stages of maturation in the development of a biofilm: Stage I involves inter-cellular communication and the generation of autoinducer signal molecules such as N-acylated homoserine lactone (AHL). Stage II entails an increase in microcolony size and thickness to a value of roughly 100 µm, resulting in the formation of a macro-colony (Thi et al. 2020). With biofilm maturation, the pathogen becomes more resistant to environmental stresses, such as nutrient deprivation, the host immune system, and antibiotic treatment. This stage is also closely dependent on quorum sensing and signaling molecules (Yan and Wu 2019; Jurado-Martín et al. 2021).

The final stage of biofilm development is dispersal, which includes release of the planktonic form of the bacteria and micro-colonies from the cavity of biofilm and its matrix. There are number of factors affecting the dispersal of a biofilm, such as changes in pH, nutrients, oxygen availability and Nitrogen Oxides (NO). One such example is the observation that an increase in glucose levels leads to a decrease in intracellular C-di-GMP, triggering a subsequent increase in flagella production and the initiation of biofilm dispersal. C-di-GMP is a signaling molecule that aids in the transition from the free-floating planktonic form to more resilient, sessile form that enables biofilm formation as well as dispersal(Valentini and Filloux 2016). It has been shown that the *bdlA* gene product is crucial for the detection of the environmental stimuli that causes *P. aeruginosa* biofilm dispersal. The BdlA protein is a chemotaxis regulator that affects the intracellular level of c-di-GMP (Gjermansen et al. 2006). *P. aeruginosa* biofilms exist in a variety of physiological states, depending on their spatial location. Pyoverdine synthesis genes, rhamnolipid synthesis genes, and quorum-sensing genes, for example, have all been found to be expressed particularly in the stalk region of mushroom shaped structures in *P. aeruginosa* biofilms (Harmsen et al. 2010; Rasamiravaka et al. 2015).

CsrA, a key RNA-binding protein, inhibits biofilm formation, by influencing the stability of mRNA transcripts encoding key polysaccharides that constitute the extracellular biofilm matrix. CsrA promotes motility rather than biofilm formation, by stabilizing transcripts encoding the main regulator of flagella, FlhDC, by regulating GGDEF/EAL encoding proteins that control c-di-GMP levels. The quorum sensing mechanism regulates the biofilm formation at various stages of their development (MartÃ­nez and Vadyvaloo 2014; Yan and Wu 2019).

### Exopolysaccharides

Psl is a multifunctional exopolysaccharide, typically comprising of D-glucose, D-mannose and L-rhamnose monosaccharides. Psl is required for the attachment of the sessile bacteria especially in non-mucoid strains, and aids in maintaining structural integrity of the biofilm. The psl (polysaccharide synthesis locus) consists of 15 genes, 11 of which are required for the synthesis and export of psl polysaccharide (Harmsen et al. 2010; Colvin et al. 2012a; Liu et al. 2025). The CsrA homolog RsmA, binds to psl mRNA and causes it to fold into a secondary stem-loop structure that blocks the SD sequence, inhibiting ribosome access and protein translation as well as biofilm formation (Colvin et al. 2012b).

Pel is composed of partially deacylated 1,4-linked N-acetylglucosamine and N-acetylgalactosamnine with the protein products of operon *pelABCDEFG* proving to be of importance in the initial attachment of the biofilm, polysaccharide synthesis and transport.

The transcription of the pel and psl genes in *P. aeruginosa* is regulated by cyclic-di-GMP, which also influences their biosynthesis. FleQ, a transcriptional regulator, represses the expression of the pel and psl operons (Harmsen et al. 2010). Certain isolates of *P. aeruginosa*, known as rugose small colony variants (RSCVs), exhibit hyper-adherence and hyper-aggregation due to increased expression of the Psl and Pel polysaccharides. These RSCVs are often associated with patients with cystic fibrosis. They are characterized by elevated levels of cyclic-di-GMP, and reducing cyclic-di-GMP levels can suppress the RSCV phenotype (Harmsen et al. 2010; Colvin et al. 2012b; Thi et al. 2020).

**Fig. 3:**
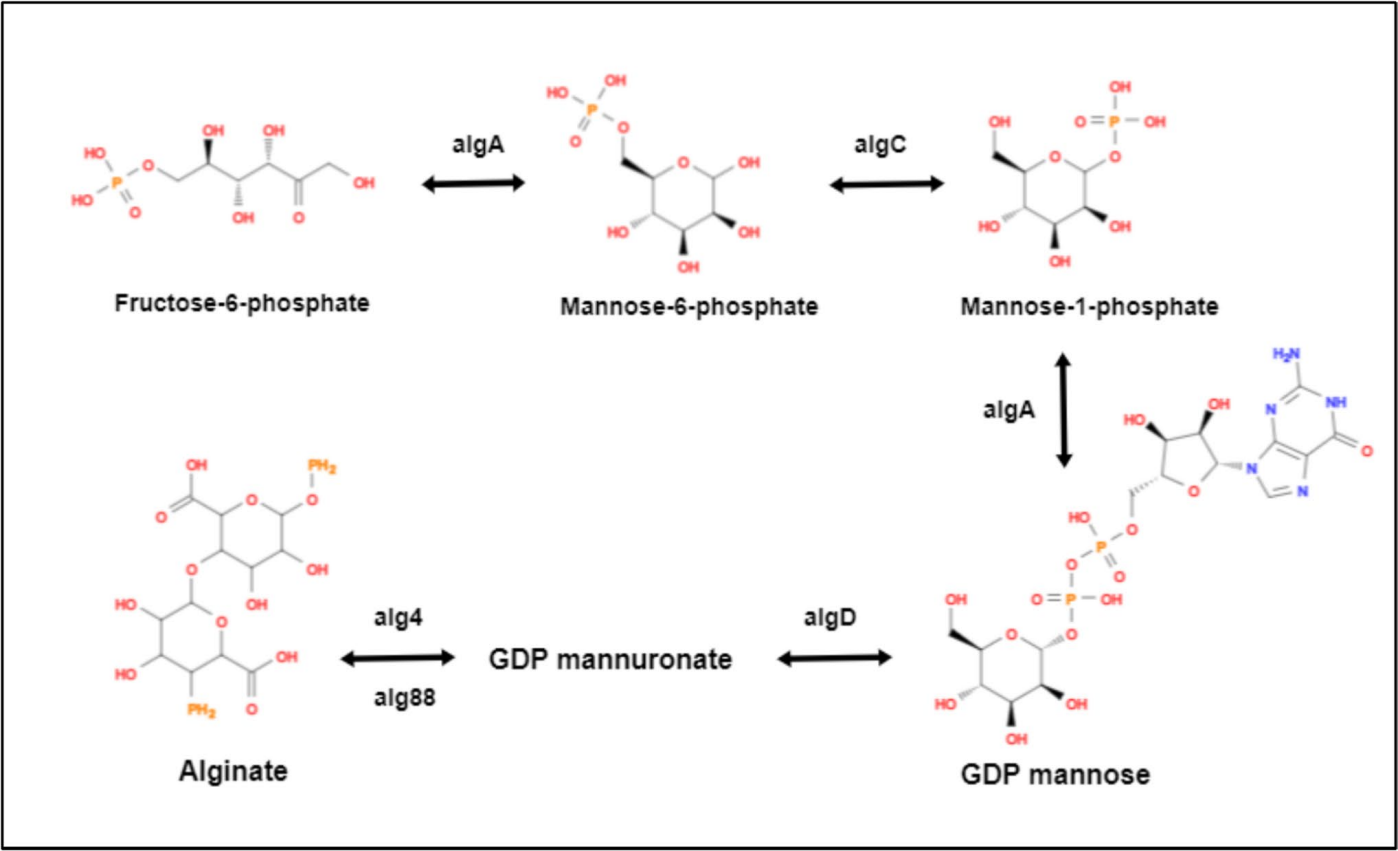
Biosynthesis of alginate with series of chain reactions facilitated by numerous enzymes and protein demonstrated in Table 1. The fructose-6-phosphate was converted to mannose-6-phosphate catalyzed by the bifunctional protein AlgA (PMI-GMP) identified as phosphomannose isomerase. AlgC is a phosphomannomutase which converts mannose-6-phosphate to mannose-1-phosphate. AlgA is GDP-mannose pyrophorylase that catalyzes mannose-1-phosphate to GDP-mannose. The continuous conversion of GDP-mannose to GDP-mannuronic acid by AlgD (GDP-mannose-dehydrogenase) modifies the reaction towards GDP-mannuronic acid and alginate production.The MucR protein, a cytoplasmic enzyme with both diguanylate cyclase and phosphodiesterase activity, is thought to produce c-di-GMP, which binds to the PilZ domain of Alg44 and triggers alginate (Moradali and Rehm 2020). The periplasmic translocation of nascent alginate is accompanied by modifications such as O-acetylation and epimerization. AlgJ and AlgX catalyse O-acetylation separately, while AlgI and AlgF supply the acetyl group donor. It has been proposed that AlgG, AlgX, and AlgK create a periplasmic scaffold for directing alginate across the periplasm, for secretion via the outer membrane protein AlgE (Schmid et al. 2015)

Alginate, which is a major anionic exopolysaccharide consists of a mixture of 1,4-linked -d-mannuronic acid (M) and its C-5 epimer, -l-guluronic acid (G). High levels of C-di-GMP promote alginate production (Rasamiravaka et al. 2015). *P. aeruginosa* alginate is naturally acetylated and is free of consecutive G residues (GG-blocks). Alginates have unique gel-forming characteristics, that make them ideal for a variety of medical and industrial applications. The structure of alginate has a significant impact upon its material qualities. With the exception of algC, thirteen proteins are directly involved in alginate biosynthesis, with the genes that encode for them being concentrated in the alginate biosynthetic operon (*algD, alg8, alg44, algK, algE, algG, algX, algL, algI, algJ, algF, algA*) (Moradali et al. 2015; Liu et al. 2025). The proteins expressed by this operon are thought to form an envelope-spanning multiprotein complex, apart from soluble cytoplasmic proteins AlgA, AlgC, and AlgD, which are responsible for delivering the activated nucleotide sugar precursor, GDP-mannuronic acid (Fig. 3**).** Alginate polymerization requires two cytoplasmic membrane-anchored proteins, the glycosyltransferase Alg8 and the putative co-polymerase Alg44 (MartÃ­nez and Vadyvaloo 2014).

**Fig. 4:**
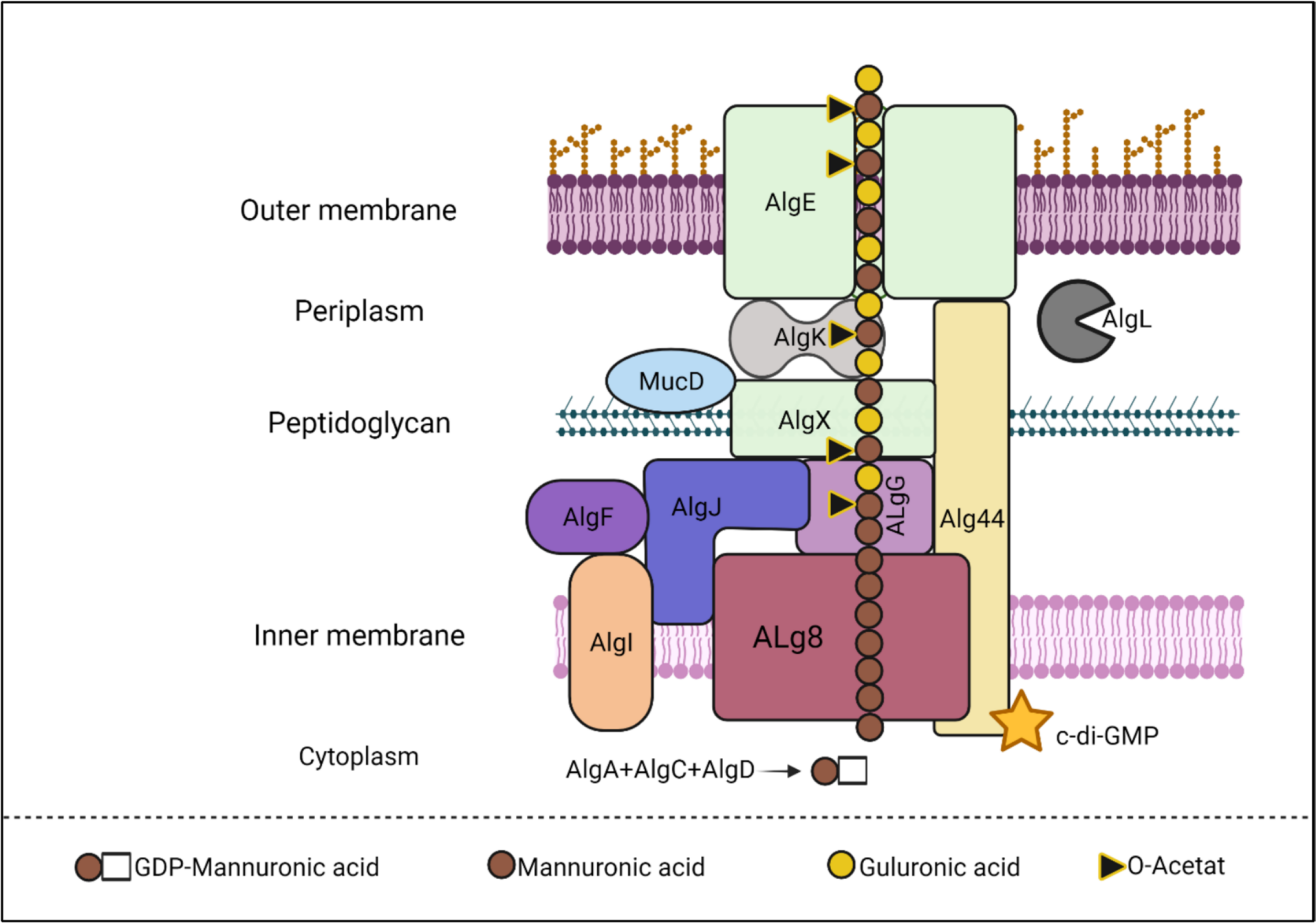
Alginate biosynthetic complex in *P. aeruginosa*. Proteins involved in polymerization are Alg8 and Alg44. The alginate lyase: AlgL and the epimerase: AlgG. The proteins involved in acetylation of what is AlgF, AlgJ and AlgI. Adapted from [149]

It has also been postulated that if alginate were accidentally delivered to the periplasm, it would be degraded by the periplasmic AlgL lyase. (Schmid et al. 2015). Previous research on protein-protein interactions and the mutual stability of proposed multiprotein biosynthetic machinery subunits, such as AlgE-AlgK, AlgX-AlgK, AlgX-MucD (a serine protease), Alg44-AlgX, and Alg8-AlgG, revealed binary protein interactions. (Jurado-Martín et al. 2021). Figure 4 illustrates the production and interaction of alginate across the pathogen. Table 3 lists the related genes and their functions.

**Table 3:** Genes involved in the regulation and production of alginate (Franklin et al. 2011)

Gene	Enzymes / Protein	Functions
algA	Phosphomannose isopmerase (PMI) GDP-mannose pyrophosphorylase (GMP) Nucleotide binding domains/ PMI-GMP	PMI Converts fructose-6-phosphate to mannose-6-phosphate. GMP converts mannose-1-phosphate into GDP-mannose.
algC	Phosphomannomutase TATA-box binding protein-like fold and mixed α/β topology	Converts mannose-6-phosphate to mannose-1-phosphate. Mannose-1-phosphate is then used to produce GDP-mannose, a key precursor for alginate production.
algD	GDP-mannose dehydrogenase	Nucleotide-binding domains
alg8	CAZy glycosyltransferase family 2	Polymerization
alg44	PilZ-like domain	Membrane-fusion protein Polymerization
algL	CAZy polysaccharide lyase family 5	Lyase
algI	Acetylase	Acetylation of polymeric mannuronic acid
algJ	SGNH hydrolase superfamily Acetylase	Acetylation of polymeric mannuronic acid
algF	Acetylase	Acetylation of polymeric mannuronic acid
algG	CASH domain Mannuronan C-5 epimerase	Epimerization of polymeric mannuronates
algK	Hydrophilic protein/ TPR-like protein	Production of protein scaffold
algX	SGNH hydrolase superfamily Hydrophilic protein without hydrophobic domain	Production of protein scaffold
algE	Β-barrel protein	Outer membrane porin channel to facilitate alginate export across the outer membrane

### Pigment

**Fig. 5:**
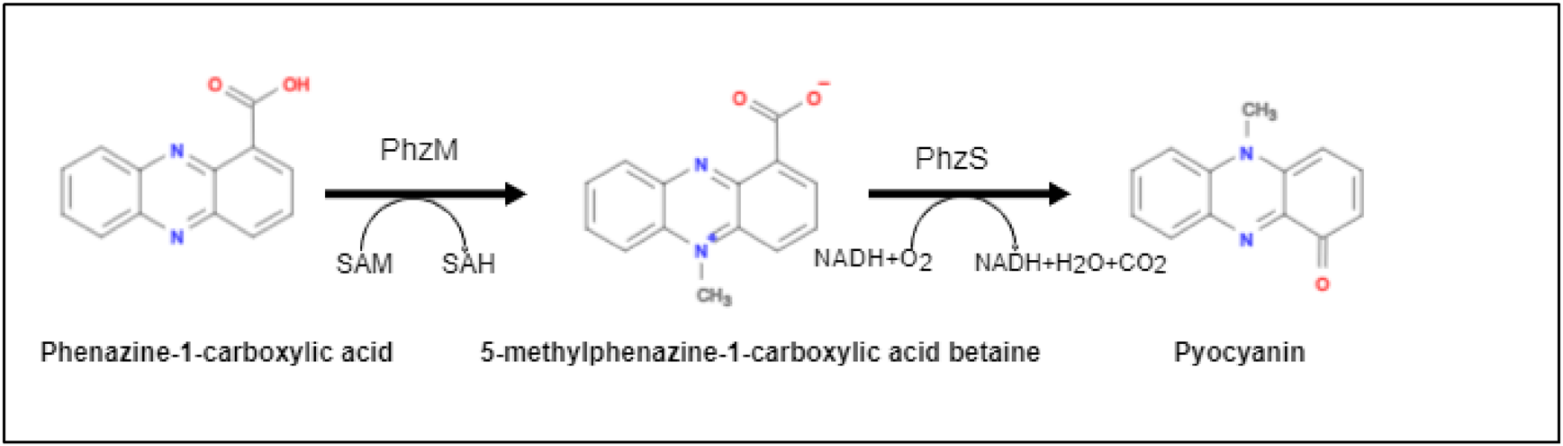
Biosynthesis of pyocyanin. Phenazine-1-carboxylic acid is transformed into 5-methylphenazine-carboxylic acid betaine by the enzyme PhzM. The product of the 5-methylphenazine-carboxylic acid betaine is further converted into pyocyanin by PhzS.

*P. aeruginosa* secretes a variety of extracellular pigments, including the phenazine molecules pyocyanin (PCN), phenazine – 1 – carboxylic acid, and phenazine – 1 – carboxamide, all of which have a high redox potential and can generate reactive oxygen species, causing harm to the host cell. Pyocyanin is a water-soluble blue-green pigment that is produced by 90-95 percent of *P. aeruginosa* strains. This is a pH-sensitive pigment, that appears blue in neutral or basic pH and turns a deep crimson or pink in acidic conditions. The production of pyocyanin involves a complex mechanism, and is encoded by several genes including phzH, phzM and phzS, as well as the operon phzABCDEFG as illustrated in Fig 5. Pyocyanin is the result of 5–methyl–7–amino–1–carboxyphenazium betaine (red) being converted into phenazine–1–carboxylic acid (yellow), which then becomes phenazine pyocyanin, via the production of S-adenosyl methionine-dependent N-methyltransferase and flavin-dependent hydroxylase. Figure 5 shows the production of pyocyanin from Phenazine – 1 – Carboxylic acid. Pyocyanin is known to cause damage to human cells via the inhibition of cell respiration, epidermal cell growth, ciliary function, disruption of calcium homeostasis (CFTR calcium channel) and causing apoptosis in neutrophils (Li et al. 2017; Nawas 2018).

**Fig. 6:**
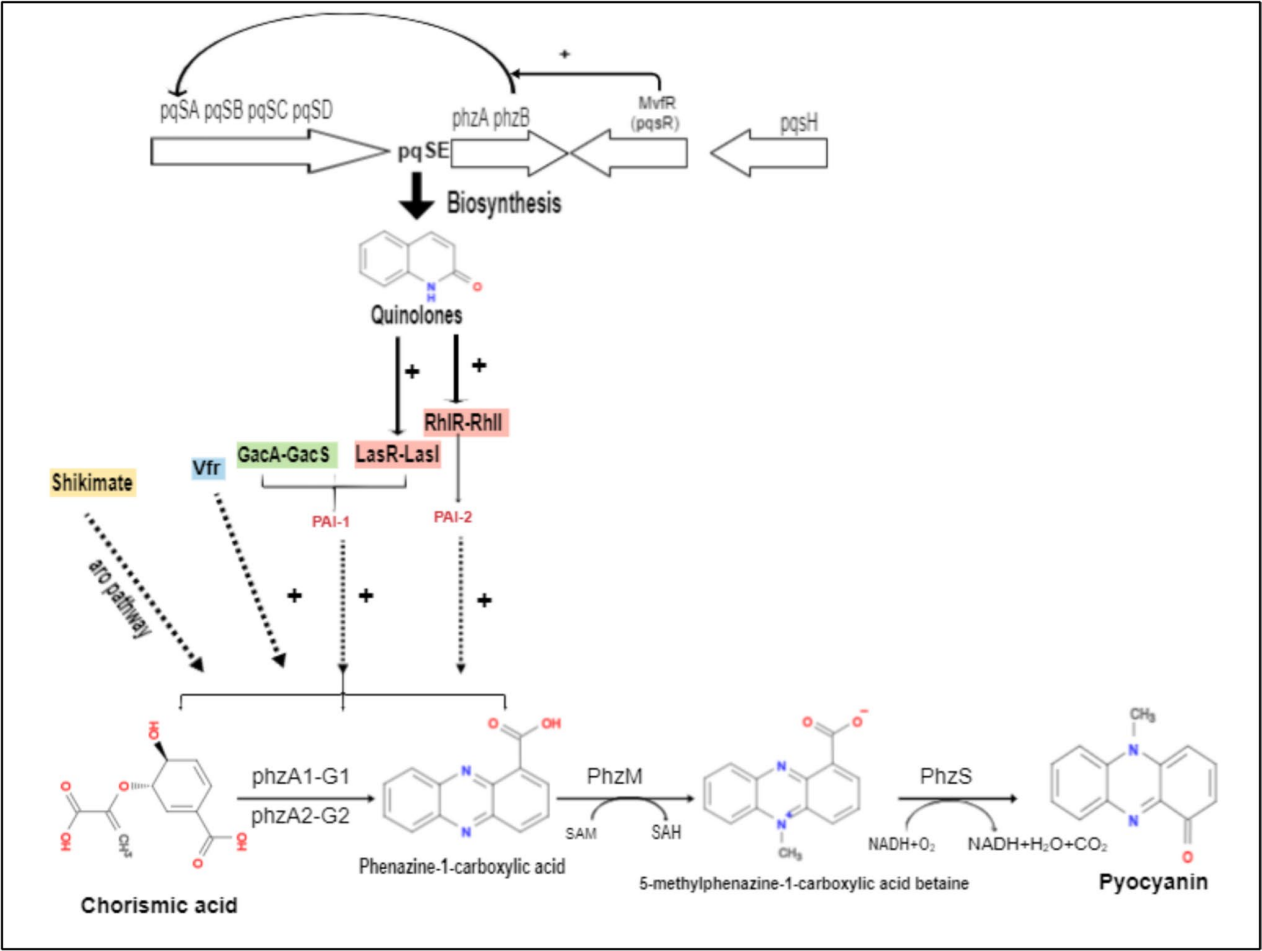
The multiple signaling systems regulating biosynthesis of pyocyanin in *P. aeruginosa*. The QS systems such as las, rhl and pqs, RhIR, and PqsE: the autoinducer synthase play an essential role in *phz* genes regulation. The *phzA1* and *phzA2* operon are in control for pyocyanin biosynthesis. The chorismic acid is converted to phenazine-1-carboxylic acid (PCA), and further into 5-methyl phenazine-1-carboxylic acid and pyocyanin pigment using *PhzM* and *PhzS* enzymes. GacA-S along with LasR-I hybrid HK functions in enhancing *phz* genes. Also, Vfr (virulence factor regulator) and the shikimate pathway is another important trigger responsible for the production of pyocyanin

Pigment production, namely pyocyanin production, is regulated by quorum sensing in *P. aeruginosa* (Fig 6). The lasI and rhlI gene products, produce N-(3-oxododecanoyl)-L-homoserine lactone (PAI-1) and N-butyryl-1-homoserine lactone (PAI-2) respectively (Nawas 2018). The transcriptional activator LasR binds to PAI-1 and RhlR binds to PAI-2 at high cell densities, to promote the transcription of virulence factor genes, several of which are implicated in PCN production. However, PCN production does not occur in if the lasR–lasI, rhlR–rhlI, and mvfR–hhq QS systems are mutated as shown in Figure 6. (Fuqua et al. 2001; Michel-Briand and Baysse 2002; Hassett et al. 2009).

### Quorum sensing

**Fig. 7:**
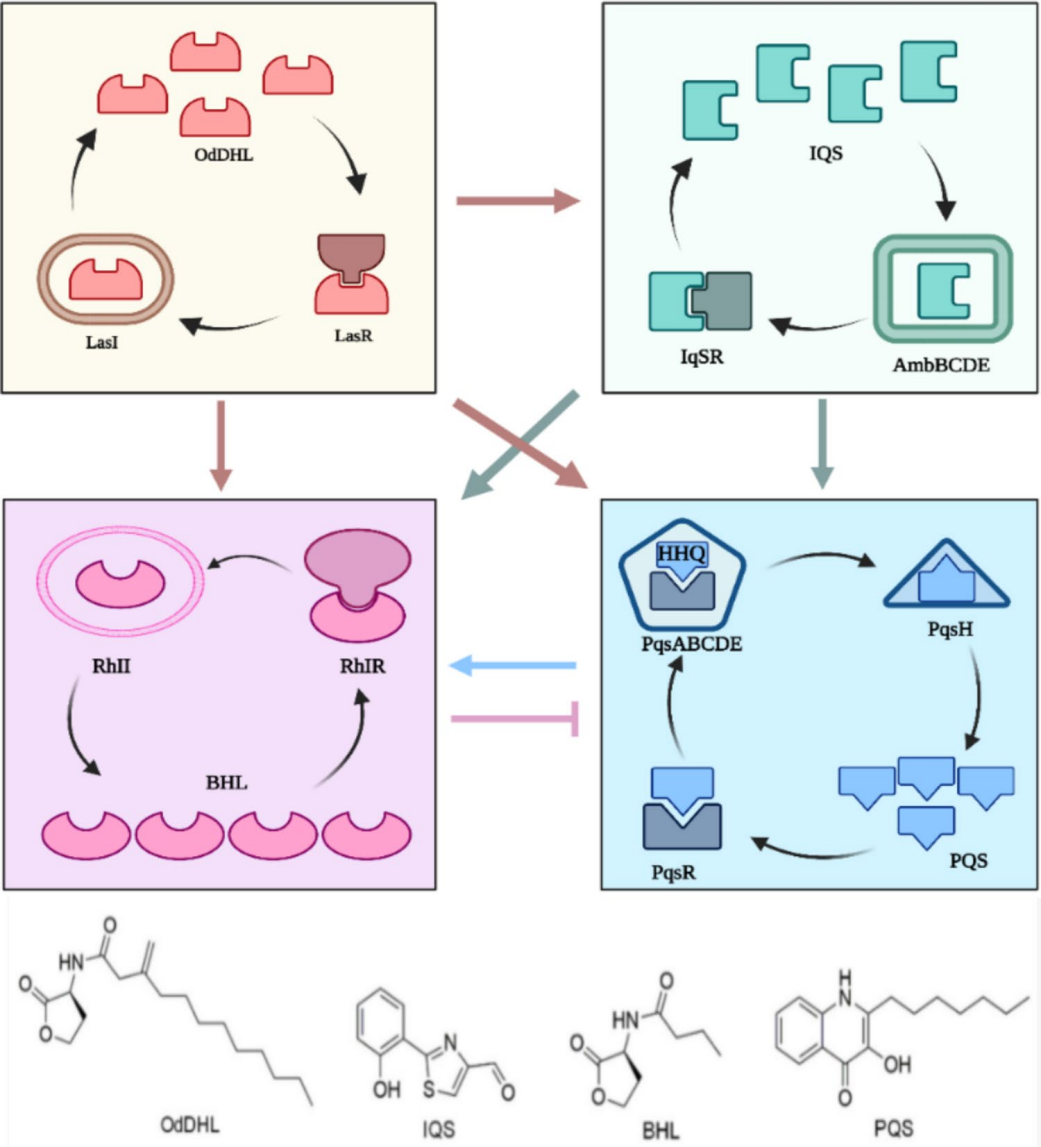
The four quorum sensing systems in *P. aeruginosa* las, iqs, rhl, and pqs and the interactions. OdDHL= N-(3-oxododecanoyl) homoserine lactone; *IQS* integrating quorum sensing signal; BHL= N-butyryl-L-homoserine lactone; *PQS* Pseudomonas quinolone signal. Positive regulation is symbolized by arrows, negative regulation by blunted arrows. Adapted from (Schütz and Empting 2018; Jia et al. 2024)

Quorum sensing (QS) is an intercellular communication system that allows bacteria to detect the density of their own cell population(Tuon et al. 2022). N-acyl-homoserine lactones in Gram-negative bacteria and oligopeptides in Gram-positive bacteria, and autoinducer molecules (AI) in both kinds of bacteria are used as the signaling molecules in QS systems. Las, Rhl, PQS, IQS represent four different routes in the QS circuits of *P. aeruginosa* that create their cognate AI (autoinducer) compounds intracellularly, N-3-oxo-dodecanoyl—homoserine lactone (OdDHL), integrating quorum sensing signal (IQS), N-butyryl—homoserine lactone (C4-HSL), 2-heptyl-3-hydroxy-4-quinolone (HHQ) respectively (Fig. 7). *The Rhl* system controls the operon rhlAB for enzyme rhamnosyltransferase (rhamnolipid production), important in biofilm formation, biofilm channel and microcolony formation, as well as detachment of cells from the biofilms, depending on the different growth stages of *P. aeruginosa*.

Quinolone-based AI and HHQ, act as ligands for the transcription factor MvfR/PqsR (multiple virulence factor), which induces transcription of number of genes, one of which codes for the enzyme cascade (PqsABCDE, PhzAB, PqsH) (Schütz and Empting 2018). The Las-Rhl QS system is involved in the regulation of PQS, LasR-3-oxo-C12-HSL which up regulates pqsABCDE promoter transcription, while RhlR-C4-HSL down-regulates it (Brindhadevi et al. 2020a)

LasR is responsible for the expression of RsaL, which is a transcriptional repressor of LasI. Binding LasR with the bidirectional Sallai promoter, inhibits the expression of both sets of genes, resulting in the generation of both positive and negative signal feedback loops to balance the levels of N-acyl HSL (Rampioni et al. 2007; Lee and Zhang 2015). According to genetic studies of numerous quorum-controlled promoters, conserved palindromic sequences known as las-rhl boxes serve as binding sites for one or both regulators (Brindhadevi et al. 2020b).

The PQS system, controlled by the autoinducer 2-heptyl-3-hydroxy-4-quinolone, is now a central target in combating antimicrobial-resistant *P. aeruginosa*, particularly for its critical role in biofilm formation and virulence. Quorum Sensing Inhibitors (QSIs) are actively being developed to target two main components: the synthesis pathway, often via enzymes such as PqsD, or the master transcriptional regulator PqsR (MvfR). Recent findings in natural product chemistry underscore the therapeutic promise of compounds like coumarin derivatives and flavonoids, which function as anti-virulence agents by antagonising PqsR or disrupting QS-regulated processes. Crucially, these natural QSIs demonstrate significant synergistic potential when paired with conventional antibiotics (e.g., ciprofloxacin, tobramycin), where the QSI disarms the pathogen and disrupts the protective biofilm, resulting in a substantial potentiation of the antibiotic's efficacy and offering a viable strategy to overcome multidrug resistance (Storz et al. 2012; D’Angelo et al. 2018; Liu et al. 2023).

Studying QS provides the critical advantage of targeting specific virulence pathways (such as PQS/PqsR) to disarm bacteria rather than kill them. This anti-virulence strategy dramatically lowers the selective pressure for resistance while restoring or boosting the efficacy of conventional antibiotics.

## Host response to *P. aeruginosa* infection

There are complex mechanisms and cell types involving the innate and adaptive immunity systems with regards to host response to infection by *P. aeruginosa*. However, most of these studies used infection models in planktonic form instead of biofilm form, as it was challenging to develop the exact same conditions in animal models (Moser et al. 2017; Maurice et al. 2018a).

The epithelium is the first line of defence, providing a physical barrier to bacterial invasion via a cell-to-cell network and tight junctions. Patients affected by disrupted epithelium, such as that caused by intubation, are at more risk of infection.

The upper respiratory tract prevents infection by the mucociliary function, by moving the upper mucus layer towards the laryngopharynx which clears 90% of inhaled particles (Ozer et al. 2005; Quinton 2010; Guzman et al. 2013). The respiratory epithelium secretes a variety of antimicrobial peptides (lactoferrin for iron chelation), proteins (defensins to kill the bacteria) to prevent biofilm formation, by stimulating the ciliary motility of the epithelium. (Hancock and Speert 2000; Boman 2003). In addition, the production of cytokine and chemokine IL-8 is increased when toll-like receptors are activated, which in turn activates cells for innate and adaptive immune system.

Cell surface receptors of the apical membrane of polarized epithelial cells such as the asialoganglioside M1 (asialoGM1), recognize the pathogen *P. aeruginosa*, resulting in activation of signal transduction pathways and the production of cytokines and chemokines (inflammatory), while also protecting the cells from invasion (Crabbé et al. 2014; Maurice et al. 2018b; Sahu and Ruhal 2025). Epithelial cells express certain enzymatic factors which play an important role in inhibiting quorum sensing molecules, and hence, fight against the pathogen's virulence by interfering with the biofilm maturation process. One such factor is the paraoxonases (PON), which use Acyl-HSL molecules as substrates to degrade the 3OC12-HSL by hydrolysing the lactone ring and hence block the quorum-sensing signalling pathway (Ozer et al. 2005).

**Fig. 8:**
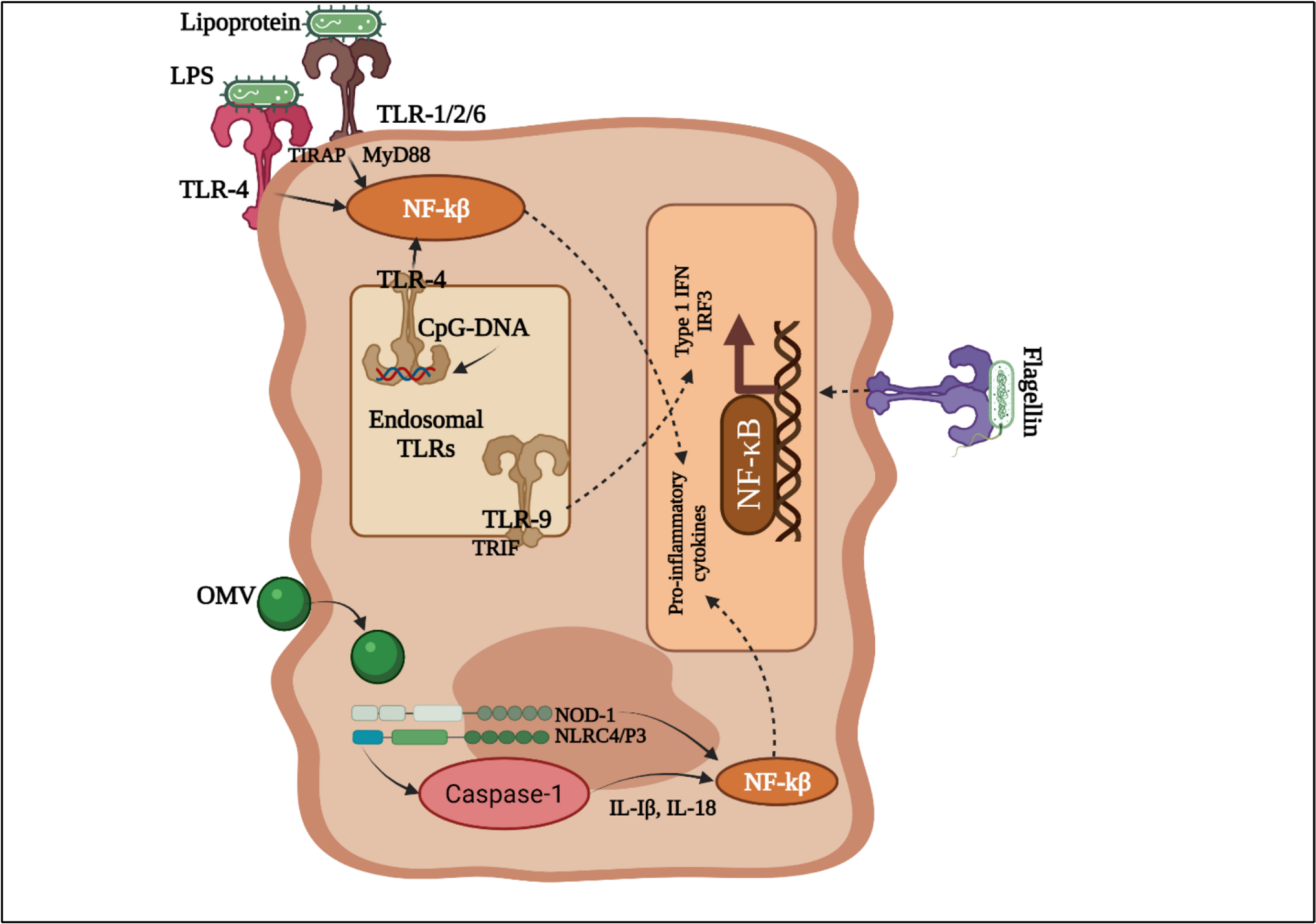
Recognition of *P. aeruginosa* in the pseudostratified respiratory epithelium. The PAMPs of *P. aeruginosa* such as flagellin, lipoprotein, LPS, and CpG-DNA are recognized by PRRs (pathogen recognition receptors) on immune cells such as TLR2, TLR4, TLR5, and TLR9 and activate pro-inflammatory cytokines and chemokines production. Outer membrane vesicle (OMV) endocytosis triggers the NF-kβ pathway

The high levels of NF-B production (Fig. 8) by CF airway epithelial cells (AECs) and the Th2-skewing of the adaptive immune response results in hyperproduction of proinflammatory cytokines such as IL-5 and IL-8 and lowers the production of anti-inflammatory cytokines such as IL-10 and INF- γ, which contribute to the pronounced IgG antibody response (Ziady and Hansen 2014; Yonker et al. 2015). While the ability of Polymorphonuclear neutrophils (PMNs’) to kill *P. aeruginosa* biofilms is compromised, the hyperinflammatory response causes major tissue injury rather than destroying the bacterium within the biofilm (Bruscia and Bonfield 2016).

**Fig. 9:**
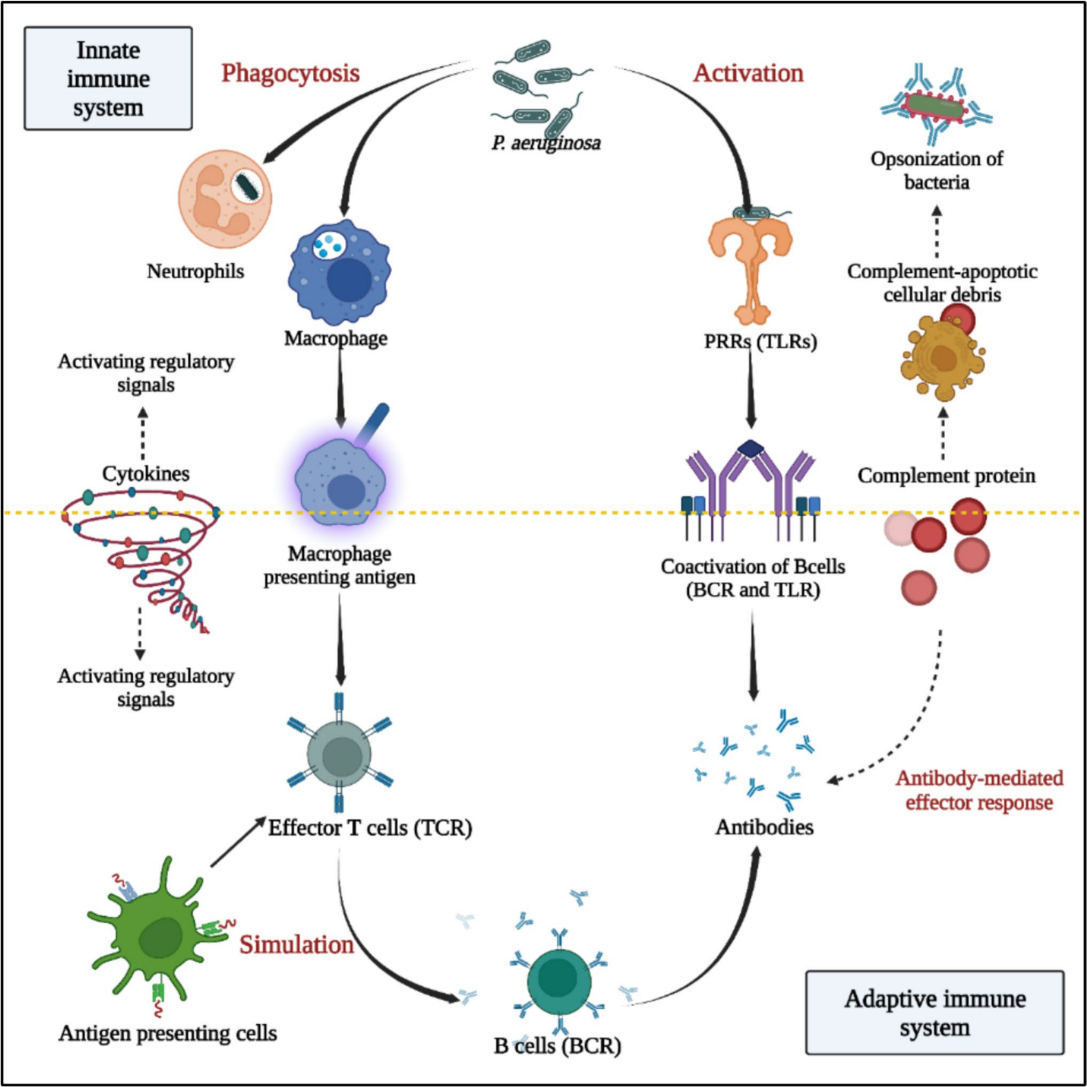
Host immune response to *P. aeruginosa*. Innate immune response presents recognition of PAMPs of *P. aeruginosa* by PRRs, stimulation of macrophages and PMNs, complement system and triggering the release of pro-inflammatory cytokines such as TNF-a, Il-1, IL-6, IL-8, and IL-12. Adaptive immune response occurs with skewed Th2 response, effector cells and activation of T-cells releasing cytokines that additionally boosts the inflammation by promoting the accumulation and activation of PMNs and IgG production. Adapted from (Eskeziyaw et al. 2020)

Innate immune cells play an important role in host defense mechanisms against pathogenic attack, by inducing an inflammatory response (Moser et al. 2017). However, *P. aeruginosa* has several mechanisms to evade destruction by neutrophils and macrophages, including regulation of rhamnolipids in the extracellular matrix, which can impair neutrophil chemotaxis. Neutrophils also present extracellular traps consisting of DNA and proteins such as neutrophil elastase, myeloperoxidase and cathepsin (Mayer-Hamblett et al. 2007). Immune response is graphically demonstrated in Fig 9.

Polymorphonuclear neutrophils (PMN) and IgG antibodies specific to *P. aeruginosa* antigens such as alginate, LPS, and proteins surround a *P. aeruginosa* biofilm, thus reinforcing the inflammatory response. *P. aeruginosa* antigens and IgG create immune complexes, which activate complement and recruit PMNs. Activated PMNs then proceed to consume oxygen and release reactive oxygen species (ROS) (Crabbé et al. 2014), proteases, and DNA, triggering an inflammatory response (Jesaitis et al. 2003). Due to the oxygen deprivation required for PMN activity, PMNs' ability to phagocytose and kill *P. aeruginosa* in sputum is impaired, leading to frustrated phagocytosis.

Macrophages and dendritic cells in particular, play a vital role in the adaptive immune response, with antigen uptake, presentation, and also function as activator cells(Sahu and Ruhal 2025) . DCs can be classified as myeloid (mDCs) or lymphoid (later plasmacytoid DCs, pDCs) based on their surface markers and cytokine responses and these DC subtypes were found to be dependent on different cytokines in their environment and to produce varied T-helper cell responses (Jensen et al. 2010). Because of their Th1-inducing potential, mDCs were classified as DC1 cells, as they produce IL-12 and depend on granulocyte-macrophage colony-stimulating factor (GM-CSF). Lymphoid DCs (later pDCs) are IL-12-deficient and IL-3-dependent cells that have been found to be activated by G-CSF and called DC2 cells because of their potential to induce Th2 responses. G-CSF, it was hypothesised, not only attracted neutrophils from the bone marrow in CF patients, but also generated DC2 cells and fostered a Th2 response (JENSEN et al. 2004). In CF patients with persistent *P. aeruginosa* lung infection, there was a significant link between the GM-CSF/G-CSF ratio, and the IFN-g response as well as lung function (Lavoie et al. 2011; Gellatly and Hancock 2013b).

Host neutrophils and macrophages (dendritic cells), are able to react to the presence of biofilms by pattern recognition receptors (PRRs), which recognize the pathogen associated molecular patterns (PAMP) and thereby activate the immune response. Flagellin and LPS, two PAMPs found in *P. aeruginosa*, bind to TLR5 and TLR4, respectively, triggering a critical but redundant pro-inflammatory signaling cascade as shown demonstrated in Fig. 8 (Raoust et al. 2009). The internalization and intracellular destruction of *P. aeruginosa*, correlates with the extracellular detection of *P. aeruginosa* by plasma-membrane associated PRRs. The cytoplasmic PRR Nod1, which detects peptidoglycan motifs, provides intracellular protection against *P. aeruginosa* (Kolbe et al. 2020).

The complement system also plays a key role in innate and adaptive immune responses by aiding the existing mechanism to combat the infection. Three major pathways include: the classical, the lectin and the alternate pathway; all of which lead to similar enzymatic signaling and converge, at C3 complement protein (found in blood) (Sarma and Ward 2011).

The classical complement pathway involves the development of immunological complexes including IgM or IgG antibodies, that bind to autoantigens. The complement C1 complex, attaches to the Fc region of the antibody, triggering a series of enzymatic cascades that produce the anaphylatoxins C3a and C5a, which subsequently bind to their receptors, and act as phagocyte chemo attractants. (Sarma and Ward 2011). Other complement by-products, such as C3b, may play a role in opsonising bacteria for phagocyte destruction. Complement activation can cause cell death by forming the membrane assault complex (C5–C9), which causes cell lysis by forming pores in the cell membrane (Lavoie et al. 2011).

When mannose-binding lectin (MBL) or Ficolin attaches to carbohydrate moieties on pathogen surfaces, the lectin pathway (LP) is triggered. MBL and ficolin both circulate in the serum as complexes with MBL-related proteins (Sarma and Ward 2011). Because they bind to the same or neighbouring epitopes on these apoptotic cells, MBL and C1q are known to compete for binding to late apoptotic blebs, activating mannose-associated serine proteases and therefore following in downstream signalling of the classical pathway (Nauta et al. 2003; Sørensen et al. 2005).

The alternative complement pathway is activated by the process known as tick over, involving hydrolysis of C3 and activating fluid phase C3 convertase, which results in binding of C3 and Factor B which in response, permits an enzyme (plasma protease) Factor D to cleave C3 into two parts namely, C3a and C3b; which is then stabilized by properdin release from neutrophils and macrophages by binding the C3b and hindering cleaving by Factors H and I. One advantage of this alternative pathway is the use of C3b from lectin or the classical pathway which can trigger the tick over process (Leinhase et al. 2006; Kemper et al. 2010).

### Impact of Biofilm on Immune System Interaction

*P. aeruginosa* biofilm formation represents a critical turning point in host-pathogen interactions, primarily by altering the immune response's ability to target the pathogen and steering the transition from acute to chronic infection. The EPS matrix, primarily made of polysaccharides (like alginate), DNA, and proteins, serves as a remarkable physical and biochemical shield against host defences (Høiby et al. 2010).

1. *Physical Impairment of Phagocytosis and Clearance*: The physical size and stability of the biofilm structure prevent immune cells, chiefly neutrophils, from effectively clearing the bacteria. This leads to a phenomenon in which neutrophils engulf bacterial microcolonies; however, they fail due to their size and the protective matrix. The neutrophils degranulate, releasing destructive reactive oxygen species and proteases into the surrounding tissue. This constant, ineffective immune attack is a primary driver of the chronic, weakening tissue damage seen in conditions like cystic fibrosis and chronic wounds (Yin et al. 2022).
2. *Evasion of Humoral Immunity and Complement:* The dense EPS matrix substantially impedes the diffusion and activity of key humoral components. Antibodies (IgG, IgA) and complement proteins strive to penetrate the deeper layers of the biofilm, preventing opsonization and complement-mediated lysis (Leid et al. 2005). Likewise, particular biofilm components, such as the mucoid polysaccharide alginate, can inhibit complement activation and mask surface antigens, making the bacteria efficiently invisible to targeted immune attack (Mulcahy et al. 2014).
3. *Shift to Chronic Inflammation:* The presence of a persistent, non-cleared biofilm persistently stimulates immune signalling pathways without resolution. This leads to a shift from a vigorous, acute inflammatory response to a state of slow, chronic inflammation. The continuous cycle of phagocytosis, ROS release, and failure to eradicate the pathogen confirms a self-disseminating inflammatory environment that promotes tissue remodeling, fibrosis, and host pathology, allowing the bacteria to persist indefinitely (Jurado-Martín et al. 2021).

*Immune response in chronic infections:* Chronic infection due to *P. aeruginosa* is associated to severe lung damage and poor clinical outcomes, driven both by immediate bacterial effects and by a persistent, dysregulated immune response. Neutrophils are the predominant immune cells, releasing proteases, reactive oxygen species, and other mediators that influence tissue injury. Other immune populations, including macrophages and specific subsets of T helper cells, play complementary roles by amplifying inflammation, releasing additional damaging molecules, or failing to maintain immune balance. An understanding of these maladaptive responses could help the development of targeted therapies to modulate inflammation and improve outcomes (Nickerson et al. 2024).

Much of the research on chronic *P. aeruginosa* infection comes from cystic fibrosis (CF), where mutations in the CFTR gene may further influence immune cell function, though the exact impact remains contested. Fewer studies have focused on other chronic lung conditions, such as COPD or non-CF bronchiectasis (NCFB) (Williams et al. 2010). The host response involves both innate and adaptive immune pathways. Airway epithelial cells and alveolar macrophages detect the pathogen via pattern recognition receptors, thereby initiating the recruitment of innate immune cells. Chronic infection is characterised by prolonged neutrophil infiltration, during which neutrophils contribute to lung injury through degranulation, the formation of neutrophil extracellular traps (NETs), and cytokine release (Ratner and Mueller 2012). Macrophages, both resident and recruited, adopt a pro-inflammatory phenotype and secrete cytokines that amplify the inflammatory milieu. Phagocytosis by neutrophils and macrophages is often impaired due to bacterial adaptations within the chronic infection environment (Williams et al. 2010).

Adaptive immunity is activated as dendritic cells present bacterial antigens to T and B cells in local lymph nodes. CD4+ T cells primarily differentiate into Th2 and Th17 populations. Th2 cells support B-cell responses and antibody production, including IgG and IgA, which often fail to clear infection effectively and may exacerbate inflammation through immune complex formation. Th17 cells produce IL-17 cytokines that promote neutrophil recruitment and further contribute to tissue damage, creating a cycle of persistent inflammation and lung injury (McIsaac et al. 2012).

In chronic *P. aeruginosa* infection, biofilms have an impact not only on bacterial survival but also on the modelling of host immune responses. Rather than protecting bacteria from antibiotics and immune clearance, biofilm communities actively influence immune function, promoting a state of persistent yet ineffective inflammation. Components of the biofilm, including exopolysaccharides, extracellular DNA, and quorum-sensing signals, interfere with conventional immune signalling, leading to continued neutrophil recruitment and activation. These neutrophils release proteases, reactive oxygen species, and form extracellular traps, causing tissue injury while failing to eliminate the bacteria. Similarly, macrophages and other immune cells exhibit impaired phagocytic activity and altered cytokine production within the biofilm environment. Concurrently, these interactions establish a feedback loop in which chronic inflammation damages host tissue but does not resolve infection, allowing *P. aeruginosa* to persist and maintain pathogenicity over time (Sahu and Ruhal 2025).

## Novel treatment strategies against *P. aeruginosa*

**Fig. 10:**
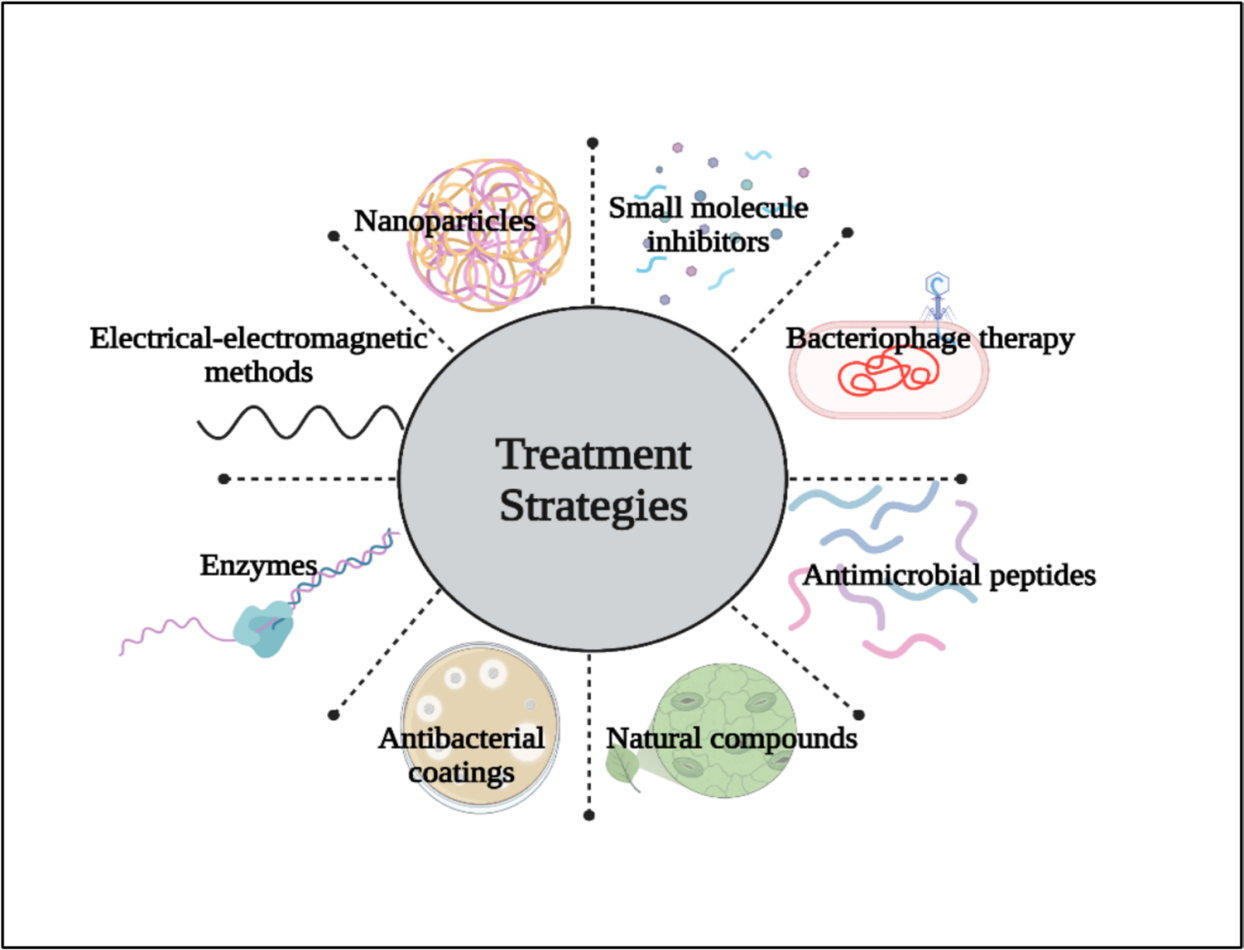
Novel treatment strategies against *P. aeruginosa*

Due to the weakening effectiveness of traditional antibiotics against multi-drug resistant *P. aeruginosa* biofilms, the circumstances of novel therapies is shifting toward strategies that prioritise neutralising the pathogen rather than directly killing it, and exploiting synergistic combination approaches. Common themes across these strategies include: 1) Biofilm Disruption, gained through agents such as enzymes and nanoparticles to break down the protective extracellular polymeric substance 2) Virulence Quenching, using compounds or enzymes to inhibit QS and toxin production; and 3) Host-Directed Therapies (HDTs), which modulate the host immune response (e.g., NETs) to decrease collateral tissue damage (Fig.10).

*Natural compounds:* With the lag in discovery of new antibiotics active against multidrug-resistant bacteria, it is vital to focus more attention on the application of natural compounds and their potential antibacterial and/or antibiofilm activities. Skyllamycins B produced by *Streptomyces sp.* showcases antibiofilm activity against *P. aeruginosa* and Promysalin produced by *Pseudomonas* auto inhibits pyoverdine production. Simplified analogues of bromoageliferin inhibits antibiofilm activity against *P. aeruginosa* PAO1 (Huigens et al. 2007; Melander et al. 2020). Similarly, a recent study researched potential application of *Plectranthus amboinicus* leaf extracts as an antibacterial and antibiofilm agent. Several classes of bioactive phytochemicals such as mono-, di-, tri-, and sesquiterpenoids, alcohols, flavonoids, esters, phenolics, phenylpropanoids, fatty acids, and aldehydes are all reported to be present in the leaves of this species (Sawant et al. 2023).

*Electrical and Electromagnetic methods:* Electroporation, which is widely investigated in combination with electromagnetic fields for the inactivation and treatment of pathogens, was defined as the application of electric fields that create stress and result in membrane disintegration (Wu et al. 2004). In order to achieve the best results, this technique is combined with other treatments. For instance, it's frequently combined with the host immune system using low AC currents, or paired with a drug such as chloramphenicol alongside extremely low-frequency electromagnetic waves (Giladi et al. 2008; Freebairn et al. 2013). These investigations support conclusions regarding the treatment of planktonic and biofilm infections, but further research is needed to determine its applicability in the clinical setting (Sawant et al. 2023).

*Antibacterial coatings:* With the widespread use of synthetic implants in surgeries, the approach is to coat the implants with antibacterial or antibiofilm agents such as Hydroxyapatite-antibiotic based coatings, photoactive coatings, and antiseptic coatings to reduce planktonic cell attachment and biofilm formation (Veerachamy et al. 2014; Khatoon et al. 2018). In a previous study, silver ions were integrated into zeolite type LTA, this would allow the release of silver ions in continuous and controlled manner. The silver-zeolite LTA (Ag-LTA) was analyzed for its antimicrobial activity by exposing borehole and well water samples from Vui, Ghana. The results showed that the Ag-LTA was effective, and the samples were totally devoid of microbes (B. Kwakye-Awuah 2021).

*Nanoparticles:* Silver nanoparticles (Ag-NPs) have demonstrated the ability to combat pathogens through multiple mechanisms, including uptake by mammalian cells (phagocytosis), disruption of microbial cell walls, and the generation of reactive oxygen species via lysosomal degradation. Nanoparticles have gained significant recognition in medical applications due to their antimicrobial properties and inherent stability, which is essential for effective drug delivery. One practical application is the surface coating of Ag-NPs on dental implants, which not only enhances antimicrobial protection but also improves biocompatibility and overall implant quality*.* (Alcudia et al. 2022). Frthermore, Ag-NPs, Cu-NPs, ZnO-NPs, and TiO_2_-NPs altered the expression levels of oxidative stress-associated genes in *Escherichia coli*, *Bacillus cereus* and *Staphylococcus epidermidis* (Metryka et al. 2022; Abdel-Fatah et al. 2024).

*Antimicrobial peptides (AMPs):* Antimicrobial peptides are a vital component for the innate host defense mechanism, and are known to have no definite mechanism, which minimizes the chance of pathogens developing resistance. However, pH and salt sensitivity and high cost of production limits the use of peptides. (Aoki and Ueda 2013). *S pyocins* are heat-sensitive proteases with antibiofilm activity against *P. aeruginosa* infection. D enantiomer of LL-37, cathelicidin in humans have shown to inhibit attachment of Pseudomonas and decrease the biofilm (Dean et al. 2011; Kapoor et al. 2011).

*Enzyme based therapies:* Alginate lyase combined with antibiotic Gentamicin is used for the successful eradication of mucoid strains of the pathogen *P. aeruginosa* especially in Cystic Fibrosis patients. (ALKAWASH et al. 2006). Paraoxnases (enzymes produced by the host immune system), which have lactonase activity, degrade acyl-homo-serine-lacton molecules of the pathogen and thereby disrupt the QS system (Ozer et al. 2005).

*Inhibition of Quorum sensing:* The periplasmic enzyme PvdQ acylase, hydrolyses N-acyl homoserine lactone, and thereby directly affects QS mechanism. M64 a benzamide-benzimidazole compound enhances the antibiofilm properties of meropenem and tobramycin by inhibiting the QS controller. MvfR. AqdC, a quorum quenching enzyme discovered recently, functions by reducing pyocyanin and alkyl quinolone production, whilst at the same time increasing elastase production (Geske et al. 2005; Maura and Rahme 2017; Utari et al. 2018; Birmes et al. 2019). The las and rhl systems in *P. aeruginosa* are targeted by halogenated furanones derived from natural chemicals, which reduce biofilm formation (Hentzer 2003).

*Bacteriophage Therapy:* The increasing prevalence of MDR *P. aeruginosa* has urged researchers to investigate bacteriophage therapy as an innovative alternative to traditional treatments. Early Phase 1b/2 trials, including those evaluating intravenous WRAIR-PAM-CF1 and inhaled BX004, have established the safety and tolerability of phage therapy for the management of chronic infections, especially in patients with cystic fibrosis. A prominent advancement in this field is the development of tailored medicine, where lytic phages are specifically selected to target an individual’s bacterial strain. This approach has shown success in studies using inhaled phages to treat MDR and PDR P. aeruginosa in CF, leading to decreased bacterial load and improvements in lung function (FEV1). Additionally, these phages can drive the evolution of bacterial mutants that are either less virulent or become susceptible to standard antibiotics again. The combination of phages and antibiotics, known as Phage-Antibiotic Synergy (PAS), has been shown to enhance bacterial eradication and improve biofilm penetration. Mechanistic studies of PAS, including those examining temperate phages that influence the lysis-lysogeny balance, indicate potential to lower the Minimum Inhibitory Concentration (MIC) of important antibiotics such as polymyxin B, thereby broadening the therapeutic applications of phages for difficult-to-treat infections. (National Institute of Allergy and Infectious Diseases (NIAID); Chan et al. 2025; Fatima and Hynes 2025).

Study by Wannigama DL, et al., demonstrates that the intranasal delivery of the therapeutic phage KPP10 is considerably more effective than systemic intraperitoneal (IP) administration for treating *P. aeruginosa* lung infections. By delivering the phage directly to the site of infection, the intranasal route accomplishes larger localization within the bronchoalveolar compartment and upholds bacterial clearance in chronic models, whereas IP delivery resulted in bacterial rebound after 14 days. Likewise, intranasal administration prevents the significant systemic antibody responses (IgG, IgM, and IgA) triggered by IP injection, thereby minimising the risk of immune-mediated neutralization of the phages. The research indicates that local respiratory delivery maximizes therapeutic efficacy and survival while evading the immunological barriers associated with systemic phage therapy (Wannigama et al. 2025).

*Neutrophil Extracellular Traps (NETs):* Recent multi-omics analyses have shown that challenges caused by the host-mediated response, especially involving Neutrophil Extracellular Traps (NETs) and oxidative stress, are an important new target for treating *P. aeruginosa* pneumonia. While NETs can help defend against infection initially, when excessive oxidative stress leads to unnecessary or poorly controlled NETs, they can harm the body instead. This harmful host response is now recognised as a key biomarker of disease severity, as the over-release of these DNA-protein complexes (NETs) causes significant collateral tissue damage, inflammation, and microvascular dysfunction. Subsequently, developing therapeutic agents that exclusively inhibit or modulate excessive NETosis signifies a promising host-directed strategy. By targeting the underlying hyper-inflammatory response rather than only focusing on antibacterial treatments, this approach may decrease lung tissue injury and support advanced clinical outcomes by preserving pulmonary function (Lin et al. 2025).

## Limitations and future perspectives

Although this review offers a broad overview of virulence mechanisms, host-pathogen dynamics, and new therapeutic approaches targeting *P. aeruginosa*, several limitations should be noted. Much of the current research relies on in vitro systems or animal models of infection, which cannot completely mimic the intricate nature of chronic, biofilm-related infections found in humans, especially those with cystic fibrosis or compromised immune systems (Jurado-Martín et al. 2021). Furthermore, the translational gap between laboratory findings and clinical application remains significant, as many promising therapeutic approaches, such as quorum-sensing inhibitors, phage–antibiotic combinations, and host-directed therapies, are still in the preclinical or early clinical stages (Fatima and Hynes 2025).

Fundamentally, the clinical reality frequently involves polymicrobial infections; the presence of co-infecting/opportunistic pathogens (e.g., *Staphylococcus aureus* or *Candida* species) intensely alters the biofilm matrix, metabolic state, and host immune signalling, demanding future therapy development to focus on broad-spectrum anti-biofilm agents or combinations optimized for these complex microbial consortia (Sibley et al. 2008).

In order to accelerate progress, future research should prioritise two currently under-researched areas: 1) Host-Directed Therapies (HDTs) targeted at controlling the dysregulated chronic inflammatory response (e.g., suppressing excessive NETosis) rather than just affecting the bacteria; and **2)** Developing real time imaging, non-invasive, and diagnostic tools to quantify biofilm burden and therapeutic penetration *in vivo* (Thom and D’Elia 2024).

The heterogeneity of bacterial strains, variations in host immune responses, and the evolving landscape of antimicrobial resistance further complicate the reproducibility and generalisation of these findings. Future studies should therefore aim to validate these findings using clinically relevant biofilm models**,** multi-omics approaches**,** and longitudinal clinical trials that evaluate the safety, efficacy, and synergistic potential of novel therapeutic strategies.

## Conclusions

For years, *P. aeruginosa* has been a significant concern in the medical community and has also served as a model organism for studying microbial pathogenesis, adaptation to various host habitats, and mechanisms of antimicrobial resistance. The ability of *P. aeruginosa* to develop resistance to several classes of antibiotics and its’ ever-increasing prevalence in the global healthcare system has made *P. aeruginosa* a major topic of clinical interest. This monograph has focused on our current understanding of the nature of *P. aeruginosa* by combining historical and recent advances, which have enabled us to interpret the underlying mechanisms responsible for its persistence and antimicrobial resistance. Furthermore, the ongoing spread of antibiotic resistance has necessitated the development of novel treatment strategies and appropriate antimicrobial therapy to manage bacterial infections and control resistant strains. This review provides a holistic overview of the different virulence factors, such as motility, biofilm formation, pigment production, and quorum sensing, that can be targeted in this pathogen. The intricate interaction between *P. aeruginosa* and the host immune system underscores the challenges posed by biofilm-associated infections, which differ significantly from those in planktonic models. Recognition of *P. aeruginosa* PAMPs by PRRs such as TLR2, TLR4, TLR5, and TLR9 triggers a cascade of innate immune responses, including cytokine production and macrophage activation, which is further amplified by adaptive immune mechanisms, such as T-cell activation and IgG production. The rise in antimicrobial resistance (AMR) worsens these challenges, driving the need for new treatment strategies. The review also highlights the importance of leveraging natural compounds, such as Plectranthus amboinicus extracts, and advanced technologies, including electroporation, nanoparticles, and antibacterial coatings, to combat biofilm-associated infections and antimicrobial resistance. These approaches, alongside enzyme-based therapies and quorum-sensing inhibitors, disrupt biofilms, improve antibiotic efficacy, and offer promising alternatives to traditional treatments. This review aims to guide future studies in developing an improved panel of antimicrobial agents that target multiple resistance mechanisms and virulence factors to effectively control infections caused by *P. aeruginosa*.

## Supplementary Information

Below is the link to the electronic supplementary material.

Supplementary file1 (DOCX 23 KB)

## Data Availability

It’s a review article, therefore there is no primary data to be included.

## Abbreviations

AHL: N-acylated homoserine lactone
Ag-LTA: Silver-zeolite LTA
AMPs: Antimicrobial peptides
AMR: Antimicrobial resistance
CF: Cystic fibrosis
COPD: Chronic obstructive pulmonary disease
EPS: Extracellular polymeric substances
GM-CSF: Granulocyte-macrophage colony-stimulating factor
HK: Histidine kinase
HAIs: healthcare-associated infections
HEMTs: CFTR modulator therapies
ICU: Intensive care unit
IE: Infective endocarditis
MBL: Mannose-binding lectin
MDR: Multi-drug resistance
PAMPs: Pathogen-associated molecular patterns
PCN: Pyocyanin
PHE: Public Health England
PMN: Polymorphonuclear neutrophils
PRRs: Pattern recognition receptors
QS: Quorum sensing
RND: Resistance-nodulation-division
RR: Response regulator
TCSs: Two-component systems
TLR2: Toll-like receptor 2
TLR4: Toll-like receptor 4
TLR5: Toll-like receptor 5
WHO: World Health Organization
