# Supplementary material for: Pseudomonas aeruginosa: associated pathogenesis, epidemiology, resistance mechanisms, host response and emerging treatment strategies
Source: Arch Microbiol. 2026 Apr 24;208(7):351. doi: 10.1007/s00203-026-04839-0 (PMC13109213; doi:10.1007/s00203-026-04839-0)
Supplement: Supplementary file 1 — Supplementary file1 (DOCX 23 KB) [file 203_2026_4839_MOESM1_ESM.docx]

1. **AHL**: N-acylated homoserine lactone
2. **Ag-LTA**: Silver-zeolite LTA
3. **AMPs**: Antimicrobial peptides
4. **AMR**: Antimicrobial resistance
5. **CF**: Cystic fibrosis
6. **COPD**: Chronic obstructive pulmonary disease
7. **EPS**: Extracellular polymeric substances
8. **GM-CSF**: Granulocyte-macrophage colony-stimulating factor
9. **HK**: Histidine kinase
10. **ICU**: Intensive care unit
11. **IE**: Infective endocarditis
12. **MBL**: Mannose-binding lectin
13. **MDR**: Multi-drug resistance
14. **PAMPs**: Pathogen-associated molecular patterns
15. **PCN**: Pyocyanin
16. **PHE**: Public Health England
17. **PMN**: Polymorphonuclear neutrophils
18. **PRRs**: Pattern recognition receptors
19. **QS**: Quorum sensing
20. **RND**: Resistance-nodulation-division
21. **RR**: Response regulator
22. **TCSs**: Two-component systems
23. **TLR2**: Toll-like receptor 2
24. **TLR4**: Toll-like receptor 4
25. **TLR5**: Toll-like receptor 5
26. **WHO**: World Health Organization
